# Exploring EEG Effective Connectivity Network in Estimating Influence of Color on Emotion and Memory

**DOI:** 10.3389/fninf.2019.00066

**Published:** 2019-10-09

**Authors:** Meei Tyng Chai, Hafeez Ullah Amin, Lila Iznita Izhar, Mohamad Naufal Mohamad Saad, Mohammad Abdul Rahman, Aamir Saeed Malik, Tong Boon Tang

**Affiliations:** ^1^Centre for Intelligent Signal and Imaging Research, Department of Electrical and Electronic Engineering, Universiti Teknologi PETRONAS, Seri Iskandar, Malaysia; ^2^Faculty of Medicine, Royal College of Medicine Perak, Universiti Kuala Lumpur, Ipoh, Malaysia; ^3^Asia Pacific Neuro-Biofeedback Association, Singapore, Singapore

**Keywords:** achromatic, color, effective connectivity network (ECN), electroencephalography (EEG), emotion, graph theory, long-term memory (LTM) retention

## Abstract

Color is a perceptual stimulus that has a significant impact on improving human emotion and memory. Studies have revealed that colored multimedia learning materials (MLMs) have a positive effect on learner’s emotion and learning where it was assessed by subjective/objective measurements. This study aimed to quantitatively assess the influence of colored MLMs on emotion, cognitive processes during learning, and long-term memory (LTM) retention using electroencephalography (EEG). The dataset consisted of 45 healthy participants, and MLMs were designed in colored or achromatic illustrations to elicit emotion and that to assess its impact on LTM retention after 30-min and 1-month delay. The EEG signal analysis was first started to estimate the effective connectivity network (ECN) using the phase slope index and expand it to characterize the ECN pattern using graph theoretical analysis. EEG results showed that colored MLMs had influences on theta and alpha networks, including (1) an increased frontal-parietal connectivity (top–down processing), (2) a larger number of brain hubs, (3) a lower clustering coefficient, and (4) a higher local efficiency, indicating that color influences information processing in the brain, as reflected by ECN, together with a significant improvement in learner’s emotion and memory performance. This is evidenced by a more positive emotional valence and higher recall accuracy for groups who learned with colored MLMs than that of achromatic MLMs. In conclusion, this paper demonstrated how the EEG ECN parameters could help quantify the influences of colored MLMs on emotion and cognitive processes during learning.

## Introduction

Long-term memory (LTM) is the storage of information over an extended period in the brain. An LTM problem can lead to difficulties in learning because it affects an individual’s ability to capture new knowledge and experiences, which eventually limits oneself from learning from past mistakes and causes poor planning, judgment or decision making. Therefore, LTM retention has been an essential research topic since the 1880s due to its importance in extending the period of memory. Factors that will lead to poor learning and LTM retention are (1) a limited amount of processing capacity in working memory (WM), (2) a negative emotional state and (3) a detrimental effect on intrinsic motivation (Baddeley, 1992; Pekrun, 1992). For the problem of the limited amount of processing capacity in WM, this can be addressed by effectively selecting and encoding the captured information in WM. For this purpose, Mayer (2003) proposed a method for learning using texts and illustrations, which is called multimedia learning (ML). This method works by concurrently storing and processing both the visual (pictures) and verbal (texts) information at once in the visuospatial sketchpad and phonological loop. However, multimedia learning is still insufficient at improving LTM retention and learning due to the negligence of emotion and motivation in the method, which can negatively influence the selective attention. Then, cognitive-affective theory of learning with media (CATLM) was introduced to improve the design of multimedia learning materials (MLMs) by incorporating both emotion and motivation that facilitates learning by increasing cognitive engagement (Moreno, 2006).

Based on such theory, researchers proposed an emotional design using appealing colors (yellow, orange, pink, green, blue, and purple) and round shapes (with anthropomorphisms) (Um et al., 2012; Plass et al., 2014). The results showed that the colored-design group has a positive emotion, increased cognitive effort, and better learning outcome, compared to neutral design (achromatic colors: grayscale, black and white; and rectangular shapes), which indicates that emotion can be induced through MLMs design. Another study reported similar results (Mayer and Estrella, 2014), where colored (red and blue) materials induced positive emotion and increased cognitive effort compared to achromatic materials. This supports the Boarden-and-Build theory of positive emotion (Fredrickson, 1998).

Color has aesthetic value, and it also influences cognition, emotion, and behavior. Clarke and Costall (2008) found that cool colors (green, blue, and violet) are associated with being comfortable, relaxing, peaceful, and calming, that can reduce stress and anxiety levels. On the other hand, warm colors (red, yellow, and orange) are more arousing, which can stimulate human feelings and activate people. Neutral colors have less emotional content and, thus, less psychological impact. In a learning context, it has been reported that warm colors used in learning materials can create a learning environment that is positive and motivating that can help learners not only to have a positive perception toward the content but also to engage and interact more with the learning materials (Plass et al., 2014). As such, we followed Goldstein’s theory (Goldstein, 1942) and design rule (Wang et al., 2008) by choosing warm colors (red and yellow) to increase attention and elicit excitement and motivation, while cool colors (green and blue) are used to produce comfort and relaxation that facilitate memory storage for this study. Red is able to induce strong feelings; both positive and negative. However, if the intensity of the red color is over-stimulating, then attention could be distracted, causing a decrease in performance levels according to the Yerkes–Dodson Law (Broadhurst, 1957). Therefore, we used red in smaller amounts compared to yellow to keep increased arousal without exceeding the optimal level of arousal (demonstrated by an arousal rating within the range of 6-8).

At present, studies on the effect of colored MLMs on emotion were assessed using (1) subjective measurement — self-rating questionnaires (Um et al., 2012; Mayer and Estrella, 2014; Plass et al., 2014); and (2) objective measurement — eye-tracking (Park et al., 2015; Stark et al., 2018) and heart rate variability (Le et al., 2018; Uzun and Yıldırım, 2018), but both techniques have a lack of information regarding the brain activity during task execution; how the brain reacts to colored vs. achromatic MLMs, and perceives, processes, acquires, stores the information is still unknown. Although the effectiveness of colored MLMs on emotion and learning is accepted, none have assessed their effects on LTM retention and cognitive process during learning with neuroimaging techniques.

Several non-invasive techniques have been used in brain science, cognition and emotion research such as EEG, fMRI, and fNIRS to study cognitive (Antonenko et al., 2010) and emotional processes (Alarcao and Fonseca, 2017) continuously. However, fMRI and fNIRS are only suitable for examining hemodynamic responses (indirect measurement) and, therefore, they are not appropriate for high-frequency brain electrical activity measurement (direct measurement). Among all, EEG is the most widely used technique for brain research due to its distinct advantage — the excellent temporal resolution. Therefore, EEG is selected and used for assessing brain responses to the effects of colored MLMs on emotion and LTM retention in this study.

Over the past few years, EEG has been used for identifying the brain responses to colored stimuli of papers, lights, shapes, and images. The responses are evaluated based on the changes in (1) event-related potential, ERP (Cano et al., 2009; Palva et al., 2011; Yeh et al., 2013; Münch et al., 2014; Rokszin et al., 2015), (2) power spectral density, PSD (Yoto et al., 2007), and (3) functional connectivity (Zanto et al., 2010). Those evaluations had concluded the findings as follows: (1) blue and green lights produce a greater amplitude of ERP at left frontal (i.e., left middle frontal gyrus) than the red light, and thus it triggers the least light adaptation and activates more brain regions. Eventually, the interaction between visual information and brain response drives the executive, attentional and emotional processing (Münch et al., 2014). Another study (Yeh et al., 2013) reported that the increased attention of a participant is observed at a shorter P100 peak latency of occipital and a greater amplitude of P300 at frontal and parietal regions. Apart from color effect on attention, researchers (Cano et al., 2009) have also found that the visual color has an influence on emotional response as observed by a greater amplitude of P300 at the frontal region for colored images that convey positive emotion; (2) red color is able to induce a higher level of perception, attention, excited feelings, and less arousing effect than blue (Yoto et al., 2007) because an increment of theta power at the midline parietal and an increment of alpha at the left prefrontal cortex, left ventrolateral prefrontal cortex (VLPFC), left temporal and midline frontal was observed in the study. This contrasts with the outcome of the light-based stimuli discussed earlier which may be due to light radiance effect, as blue light has a great alerting effect (Cajochen, 2007); (3) An experimental study (Zanto et al., 2010) shows that color induces a long-range alpha phase synchronization between regions of frontal and occipital and activates prefrontal regions. Combining the observations above, EEG can be used as an excellent technique to assess the influences of colored MLMs on emotion and cognitive processes during learning.

It is well known that the regions of prefrontal, frontal, temporal, posterior association (parietal and occipital), are responsible for color processing, working memory, emotional processing and LTM storage (Zeki and Marini, 1998; Zanto et al., 2010). We hypothesized that colored MLMs (versus achromatic MLMs) increased activation in the prefrontal and frontal regions, as well as posterior association cortices with top–down interactions — connectivity between these regions representing improved working memory, emotional processing, and LTM storage resulting from anticipatory control and motivational factors. This paper is organized as follows: Section “Materials and Methods” for data acquisition, analysis, and statistical criteria. Section “Results” reports experimental results. Sections “Discussions” and “Limitations and Future Directions” discuss results as well as limitations and suggestions for future work. Section “Conclusion” concludes the paper’s findings.

## Materials and Methods

### Participants

The datasets comprised 45 healthy adults who were local Malaysian students recruited from Universiti Teknologi PETRONAS [18 females and 27 males; 18-24 years of age: mean 20.12 (±0.47)]. They were randomly assigned to one of the three groups: (1) learning material with achromatic illustrations (gray, black-and-white: GB&W); (2) learning material with cool-colored illustrations (green and blue: CCI); and (3) learning material with warm-colored illustrations (red and yellow: WCI). Both genders were equally represented in all three groups (six females and nine males each). All participants were right-handed — thus avoiding hemispheric lateralization — and physically and mentally healthy, without a history of head injury. Only non-smokers and with normal or corrected-to-normal vision (including normal color vision) were chosen. Before arrival at the laboratory, participants were prescreened using self-report questionnaires to ensure all inclusion criteria were met. The prescreening included a prior domain knowledge questionnaire (e.g., “I can explain what antibodies are.”) (Mayer, 2009; Plass et al., 2014), a short form Ishihara colorblind test (ICBT) (Ishihara, 1960), a Visual-Aural-Read/write-Kinesthetic (VARK) learning styles inventory (Fleming and Mills, 1992) and demographic information (age, gender, education level). Volunteers, who scored below eight-points for prior domain knowledge questionnaire, full scores for ICBT, and had a modality preference favoring V and R modes were invited to participate. Overall, learners’ prior knowledge was low (GB&W: *M* = 4.57, *SD* = 0.59; CCI: *M* = 4.57, *SD* = 0.40; WCI: *M* = 4.50, *SD* = 0.54; where total score is 12). There is no significant difference between groups in the prior knowledge test as determined by one-way ANOVA [*F*(2,39) = 0.006, *p* = 0.994]. Participants were also instructed to refrain from consuming caffeine for at least 8 h before experimental sessions, as both caffeine and nicotine are the psychostimulants that impact cognitive functions (attention and alertness) (Nehlig et al., 1992; Warburton, 1992). The study protocol was approved by the Medical Research Ethics Committee (MREC), UniKL RCMP. All participants signed informed consent forms after full disclosure. All were compensated monetarily for their time. Three participants were excluded from the analysis due to low recall accuracy (<40% correct rate, each from GB&W and CCI groups) and low emotional valence (from WCI group), resulting in 14 participants for each group.

### Computer-Based Multimedia Learning Material

Learning materials comprised 15 multimedia slides: an introductory slide, 13 content slides, and a final slide to recap overall content (adapted from Mayer et al., 2008; Mayer and Estrella, 2014). Each slide showed an explanatory text of 60-120 words (total word count: 1043; time per slide: 38 ± 16 s) describing the properties and characteristics of the virus and bacteria; as well as schematics illustrated the life cycle of viral infection and replication for understanding how viruses affect the cells they infect. By taking into account the recommendation by Mayer and Estrella (2014), EEG measurement was added to the subjective measurement to examine the direct measures of color’s effect on emotion and cognitive processing during learning.

**FIGURE 1:**
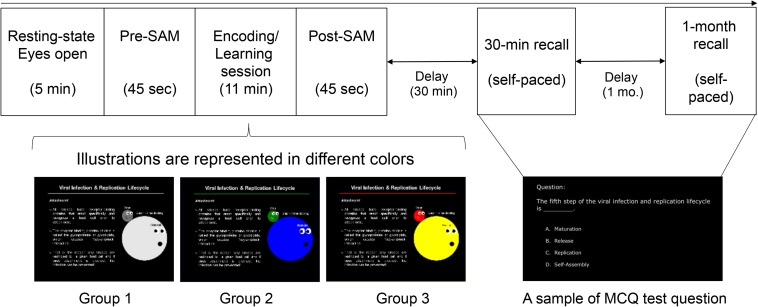
Experimental protocol and stimuli. The sequence of task sessions **(top)**, the three versions of MLMs for the learning task **(bottom)** [achromatic-colored illustrations (Group 1: GB&W); cool-colored illustrations (Group 2: CCI); and warm-colored illustrations (Group 3: WCI)], and a sample of test questions for the memory recall tests.

Biological science was used for the content slides (learning materials) that served as new knowledge for engineering students. The spatial properties (slide layout: location of text and picture) and typographies (text foreground and background, font size) were designed to be identical for all the three groups, but the visual illustrations of MLMs were displayed in either colored or achromatic versions. Specifically, the color conditions of MLMs were designed as follows: (1) achromatic colors served as control group (Group 1: GB&W); (2) cool colors for experimental condition #1 (Group 2: CCI); and (3) warm colors for experimental condition #2 (Group 3: WCI). The text was designed to be white on a black background to minimize the risk of visual fatigue (eye strain) due to viewing a computer screen for a long time (MacDonald, 1999) and it is the neutral combination. Participants rated their emotional state using SAM scale, which served as a manipulation check for the design quality affected participants’ emotion (emotion elicitation) using learning material. The screenshots of the mentioned MLMs design were depicted in Figure 1.

### Experimental Task

Participants were seated in a partially sound-attenuated air-conditioned recording room and treated individually. All of them were briefed on the experimental tasks which consisted of 7 task sessions: (1) Resting-state eyes open, (2) Pre-experiment self-rating emotion (pre-SAM), (3) Learning task, (4) Post-experiment self-rating emotion (post-SAM), (5) Distraction task, (6) Recall test after 30-min, and (7) Recall test after 1-month. EEG signals were recorded for all the seven sessions. Since the focus of this study was to evaluate the color’s effect on emotion and cognitive process during learning, only resting-state eyes open (session 1) and learning task (session 3) readings were analyzed and presented. Figure 1 (see the previous section) illustrates the experimental design. Details regarding each task session are described below.

#### Session 1: Resting-State Eyes Open

During the resting-state eyes open (EO), participants were asked to sit relaxed and quietly with hands on their thighs while looking at a white cross on a black background presented in the center of the computer screen to minimize eyeball movements.

#### Session 2: Pre-experiment Self-Rating Emotion (Pre-SAM)

Before beginning the learning task, participants were asked to rate their emotion using Self-Assessment Manikin (SAM) (Bradley and Lang, 1994). SAM is a non-verbal, pictorial technique, which directly measures levels of the emotional dimensions of valence (V), arousal (A), and dominance (D). This assessment was rated twice (before and after learning) for V, A, and D using a “9-point Likert scale,” in which the score ranged from 1 to 9 at the interval of 1 [V (1 = negative, 5 = neutral, 9 = positive), A (1 = calm, 5 = neutral, 9 = excited), D (1 = low, 5 = neutral, 9 = high)]. The self-emotional rating performed before learning served as a baseline of emotional state (pre-SAM) whereas rating performed after learning was used to indicate the emotional state while learning the materials (post-SAM). The changes in valence, arousal, and dominance (ΔV, ΔA, ΔD) between pre-SAM and post-SAM scores were assessed (see Section Statistical Analysis).

#### Session 3: Learning Task

During the learning task, participants were instructed to learn and memorize the contents of learning materials without taking notes and informed they would be tested to measure retention of the learned contents. Note-taking was not allowed due to these four reasons: (1) it requires deeper processing implies semantic encoding that facilitates learning and increases recall (encoding effect) (Bretzing and Kulhavy, 1979), (2) it interferes with visual information processing where attention was divided between the visual presentation of learning materials and his/her hand movements (Ash and Carlton, 1953; see Kobayashi, 2005 for review), (3) requires more cognitive effort and time than reading alone that depends on working memory to manage comprehension and selection of information (Piolat et al., 2005), and (4) brain controls hand movements that cause motion artifacts, all of which are likely to influence EEG signals.

#### Session 4: Post-experiment Self-Rating Emotion (Post-SAM)

Right after viewing the MLMs, participants were asked to rate their emotional response using SAM again (identical to Session 2), to indicate how they felt while studying the MLM.

#### Session 5: Distraction Task

During the retention period of 30 min, participants performed a mental arithmetic task that served as a distraction task to prevent rehearsal of learned content, by counting backward from 1000 to 265 by sevens (993, 986, 979, …, 265). This was followed by a 5-min break to ensure that the brain returned to its normal state for the recall test.

#### Session 6: Recall Test After 30-min

**FIGURE 2:**
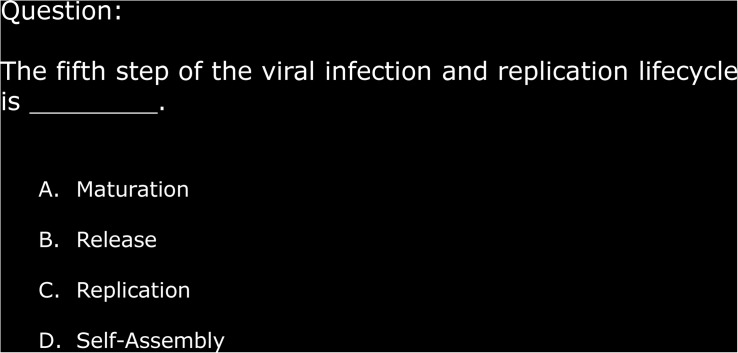
A sample of the multiple-choice questions (MCQs).

A recall test was administered after 30 min had elapsed. A recall test is commonly used to measure long-term retention of learned information. The test consisted of 15 multiple-choice questions (MCQs) covering the content of the MLMs that required participants to recall specific information (factual recall). Each test question had 4 alternatives with one correct answer. Participants were asked to answer as quickly as possible (but without time pressure) by pressing a key on the keyboard for selecting the correct answer. Reaction times (RTs) and accuracy (ACC) were recorded for behavioral data analysis. RT is defined as the time from stimulus onset to key-press response; ACC is the percentage of the correct answer. Means of RT and ACC were computed for each subject and for each group. When the test was completed, participants were instructed not to review any of the subject material from the Internet or books within the 1-month delay interval, and they allowed to leave. A sample of the 15-MCQs is illustrated in Figure 2.

#### Session 7: Recall Test After 1-Month

One month after the learning session, participants returned and took a recall test. The same questions from session 6 were used for assessing the retention of learned materials (LTM recall).

All stimuli were displayed using E-Prime 2.0 software (Psychology Software Tools, Inc., Sharpsburg, PA, United States) on a Dell laptop with a 14′′ screen (1024 × 768 pixels, 60 Hz refresh rate), at a distance of ∼30 cm from the seated participant. E-Prime software was also used to record self-report emotional experiences and behavioral responses.

### EEG Acquisition and Pre-processing

**FIGURE 3:**
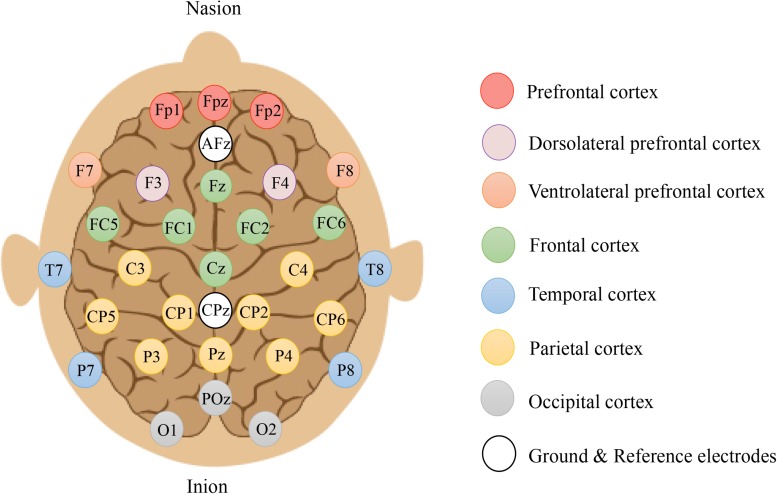
Electrode placement using the Extended International 10–20 system (10% system) covering prefrontal (red circles), dorsolateral prefrontal (purple circles), ventrolateral prefrontal (orange circles), frontal (green circles), temporal (blue circles), parietal (yellow circles), and occipital (gray circles) cortices. Odd numbers = left, even numbers = right, and z = zeros in the midline.

Scalp EEG signals were acquired using an *eegosports* amplifier (ANT Neuro, Enschede, Netherlands) with 31 gel-based Ag/Ag-Cl electrodes mounted on an EEG head cap. All electrodes were referenced to CPz and grounded at AFz according to the manufacturer’s recommendation (eemagine Medical Imaging Solutions GmbH, Berlin, Germany). The readings of the electrode’s impedance were all maintained at < 10 kΩ and sampled at 2048 Hz throughout recording sessions. Figure 3 illustrates the topographical grouping of electrodes, which are defined as: prefrontal cortex (PFC: Fp1, Fp2), midline prefrontal cortex (mPFC: Fpz), ventrolateral prefrontal cortex (VLPFC: F7, F8), dorsolateral prefrontal cortex (DLPFC: F3, F4), frontal cortex (FC: FC5, FC1, FC2, FC6), midline frontal cortex (mFC: Fz, Cz), temporal cortex (TC: T7, T8, P7, P8), parietal cortex (PC: C3, C4, CP5, CP1, CP2, CP6, P3, P4), midline parietal cortex (mPC: Pz), occipital cortex (OC: O1, O2), and midline occipital cortex (mOC: POz).

Raw EEG signals were pre-processed offline to remove unwanted artifacts using BESA Research 6.0. To eliminate the high-frequency physiological noise and low-frequency drifts, a band-pass filter was used to extract the desired band between the low- and high-cutoff frequency (0.5 and 48 Hz). Artifacts like blinking, horizontal (HEOG) and vertical (VEOG) eye movements and heartbeats were visually inspected and automatically removed via spatial filtering based on artifact and brain signal topographies using a preselection approach (Ille et al., 2002) implemented in the BESA. Artifact selection steps were involved by selecting a block epoch which contains an artifact to be used to identify individual artifact topography. This was followed by a search for further artifact occurrences and, finally, the average of all detected artifacts (e.g., blinks) were computed and marked artifact segment. The variance at ≥95% that explained by the principal component analysis (PCA) topography of the marked artifact segment is selected and the data are automatically corrected. The same steps were repeated for another kind of artifacts, such as HEOG and VEOG. To evaluate the outcome of the artifact correction, the relative root mean squared error (RRMSE) were computed to check for the overall deviations between corrected values, $\hat{X}$ and original measured values, *X* as expressed by, $R\,R\,M\,S\,E=\left(\left(R\,M\,S\,\left(X-\hat{X}\right)\right)/R\,M\,S\,\left(X\right)\right)$^∗^100% [similar approach used by De Clercq et al. (2006)]. Data with a voltage amplitude exceeding ±100 μV were rejected manually. Finally, the corrected EEG data were exported for power spectral analysis and effective connectivity analysis using custom-made scripts and open-source toolboxes in MATLAB (The MathWorks, Inc.). The open-source toolboxes included (1) EEGLAB (Delorme and Makeig, 2004) for topographical maps plotting, and (2) Brain Connectivity Toolbox, BCT (Rubinov and Sporns, 2010) for graph theoretical analysis.

### Surface Laplacian Transformation

The volume conduction effects might be overcome by source imaging algorithms such as exact low-resolution electromagnetic tomography (eLORETA), weighted minimum-norm estimation (wWNE), beamforming, etc. However, these methods have limitations. Firstly, inaccurate source reconstruction can be caused by using a fewer number of electrodes than the minimum quantity (64 electrodes) required for such source analysis (Seeck et al., 2017). Secondly, the literature (Mahjoory et al., 2017; Cai et al., 2018; Miinalainen et al., 2019) reported that there was no unique solution for the inverse problem as source reconstruction algorithms require: (1) exact inverse and forward models, (2) choices of anatomical template and head volume conductor model (conductivities for major tissues), and (3) prior assumptions of sources. On the other hand, Surface Laplacian (SL) offers to estimate the current-source density (CSD) with several advantages, including a head volume conductor model and that the assumptions of sources are not required, reference-free and only low-density electrodes (<64 electrodes) are needed (Perrin et al., 1989; Kayser and Tenke, 2006). Considering Occam’s razor principle, the SL is found suitable to be used for CSD estimation, and thus applied to the corrected EEG signals in our study, to improving topographical localization (i.e., a step to reduce the spatial spread of activity) and minimizing volume conduction effects (for a similar approach, see Lachaux et al., 1999; Johnson et al., 2017).

### EEG Frequency Decomposition

The corrected EEG signals were decomposed into frequency bands of interest using Fast-Fourier Transform (FFT), where the decomposed five frequency bands: Delta (0.5–4 Hz), Theta (4–8 Hz), Alpha (8–13 Hz), Beta (13–30 Hz), and Gamma (30–48 Hz) (Cochran et al., 1967). A 50% overlap Hanning window was used to reduce spectral leakage.

### Effective Connectivity Network (ECN)

We used the phase slope index (PSI) (Nolte et al., 2008) to estimate magnitude and direction of information flow between the multivariate EEG signals (source code for implementation of PSI is available at http://doc.ml.tu-berlin.de/causality/). PSI was employed because it is insensitive to volume conduction and it detects only non-zero phase delays that allow effective connectivity network (ECN) estimation at sensor level (Nolte et al., 2008; Mahjoory et al., 2017). Besides, the approximated fixed time delay (τ) that corresponds to a linear phase shift (in frequency domain) can be used to characterize the interacting regions of the brain. One should be noted that the phase slope of cross-spectra Φ(*f*) is also a function of frequency, which can be written as Φ(*f*) = 2π*f*τ. The sign of the phase slope is either positive or negative, which infers direction (e.g., electrodes *i* and *j*) — specifically, the causal direction from *y*_*i*_ to *y*_*j*_, if positive (*i* = sender and *j* = receiver). Otherwise, from *y*_*j*_ to *y*_*i*_, if negative. Mathematically, PSI is expressed as

$${\tilde{\Psi }}_{\mathrm{ij}}=ℑ\left(\sum_{f\in F}{\text{Coh}}_{\mathrm{ij}}^{{}^{*}}\,\left(f\right)\,{\text{Coh}}_{\mathrm{ij}}\,\left(\text{f}+\delta \,\text{f}\right)\right)$$

where $C\,o\,h_{i\,j}\,\left(f\right)=S_{i\,j}\,\left(f\right)/\sqrt{S_{i\,i}\,\left(f\right)\,S_{j\,j}\,\left(f\right)}$ is the complex coherency; *S* is the cross-spectral matrix; δ*f* is the frequency resolution (δ*f* = *F*_*s*_/*n*_*FFT*_ = 0.5Hz); ℑ(.) is the imaginary part of coherency; F is the set of frequencies over which the phase slope is summed; $\tilde{\text{Ψ}}$ is the weighted average of the slope and vanishes if the imaginary part of the coherency diminished, thus, it is insensitive to mixtures of non-interacting sources (zero phase, robust measure) (Nolte et al., 2004). Lastly, PSI values are normalized by dividing its own value with standard deviation which estimated by the Jackknife method (Nolte et al., 2008),

$$\Psi =\tilde{\Psi }/s\,t\,d\,\left(\tilde{\Psi }\right)$$

**FIGURE 4:**
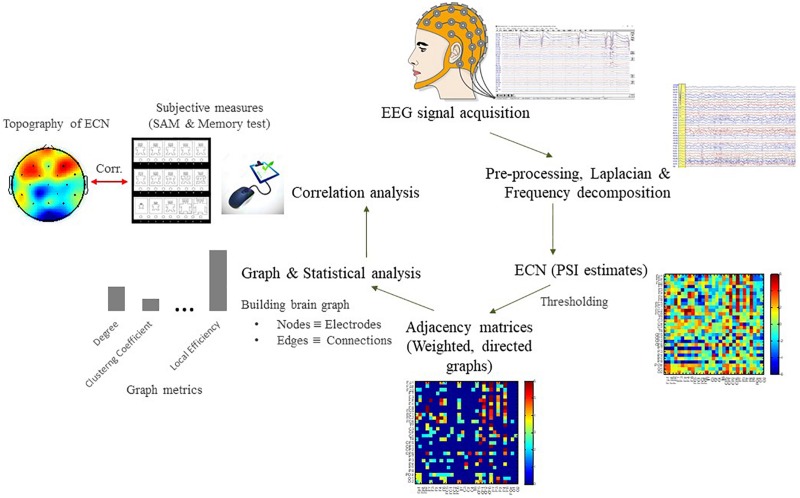
Flowchart for the EEG data analysis pipeline to characterize patterns of effective connectivity network (ECN) based on graph theoretical analysis. See Supplementary Material for results of power spectral density (PSD).

The purpose of normalization is to minimize the false positives of effective connectivity (improved specificity). The normalized PSI were stored in a 29 × 29 skew-symmetric matrix which equivalent to 406 possible pairwise associations [(*N*^2^−*N*)/2, where *N* = 29 is the number of electrodes] with diagonal being zero, so that only cross-correlation rather than auto-correlation between EEG signals are estimated. A statistical threshold (|Ψ| > 2) which corresponds to 95% confidence interval of the PSI distribution at *p* < 0.05 (two-tailed), was applied to the association matrix (Nolte et al., 2008) so that all the values below the threshold were set to zero and values above the threshold were retained its original values. This thresholding is used to generate a directed and weighted adjacency matrix for the following graph theoretical analysis. The steps of data analysis are depicted in Figure 4.

### Graph Theoretical Analysis

We charted the topological organization of ECN using graph theory. A network/graph was defined as a set of nodes (i.e., electrodes) that represent interconnected brain regions by a set of edges (i.e., entries of the adjacency matrices). Key graph metrics describing the architecture of the network were computed from the directed and weighted adjacency matrices using BCT (Rubinov and Sporns, 2010), to include node degree (k), clustering coefficient (CC), characteristic path length (λ), local efficiency (LE), global efficiency (GE), betweenness centrality (BC), and sparsity.

#### Node Degree

The degree of a node is defined as the number of edges connected to that node. Since an adjacency matrix generated from PSI values shows the direction of information flow, a node’s degree is computed by dividing it into (1) in-degree ($k_{i}^{i\,n}$) to denote incoming flow strength; and (2) out-degree ($k_{i}^{o\,u\,t}$) to denote outgoing flows strength; which are expressed as $k_{i}^{i\,n}=\sum_{j\in N}A_{j\,i}\,$ and $k_{i}^{o\,u\,t}=\sum_{j\in N}A_{i\,j}$ (where *A*_*ij*_ does not necessarily equal *A*_*ji*_ and represents the entry of the adjacency matrix). A node with high out-degree values indicates a region that could influence others. Likewise, a node with high in-degree values indicates an area influenced by other regions. Then, the total degree (TD) provides a measure of a node’s hubness which can be computed by $T\,D=\sum k_{i}^{i\,n}+\sum k_{i}^{o\,u\,t}$.

#### Directionality Index

To show directionality of information flow, the difference between out- and in-degree at each electrode was computed. Mathematically, the directionality index (DI) is expressed as $D\,I=\sum k_{i}^{o\,u\,t}-\,\sum k_{i}^{i\,n}$. A positive DI indicates that the electrode behaves like a source/sender — sending information, while negative DI means a sink/receiver — receiving information.

#### Phase Slope Index: Left vs. Right Hemisphere of Frontal-Parietal-Occipital

The mean absolute PSI were computed for different distances between anterior and posterior electrode pairs in the left and right hemispheres, respectively. The combinations were FP1/2–F3/4, FP1/2–FC1/2, FP1/2–FC5/6, FP1/2–C3/4, FP1/2–CP1/2, FP1/2–CP5/6, FP1/2–P3/4, and FP1/2–O1/2.

#### Measure of Segregation

The clustering coefficient (CC) measures network segregation; i.e., the degree to which a network is organized into local specialized regions (Watts and Strogatz, 1998). We averaged all local clustering coefficients to yield the graph’s clustering coefficient ranging from 0 to 1. Mathematically, the graph’s clustering coefficient can be written as

$$C\,C=\frac{1}{N}\,\sum_{i\in N}C_{i}=\frac{1}{N}\,\sum_{i\in N}\frac{t_{i}}{\left(k_{i}^{\mathrm{out}}+k_{i}^{\mathrm{in}}\right)\,\left(k_{i}^{\mathrm{out}}+k_{i}^{\mathrm{in}}-1\right)-2\,\sum_{j\in N}A_{\mathrm{ij}}\,A_{\mathrm{ij}}}$$

where *C*_*i*_ is the local clustering coefficient; *t*_*i*_ is the number of triangles around each node; $k_{i}^{i\,n}$ and $k_{i}^{o\,u\,t}$ is the in-degree and out-degree of a node; and *A*_*ij*_, *A*_*ji*_ is the entry of the adjacency matrix.

Local efficiency (LE) measures the efficiency of information transmission within local clusters, indicating how efficient its neighbors can communicate when a node is removed.

$$LE=\frac{1}{2\,N}(\sum_{i\,\text{N}}\frac{\sum_{\text{j},\text{h}\in \text{N},\text{j}\neq \text{i}}\left({\text{A}}_{\text{ij}}+{\text{A}}_{\text{ij}}\right)\,\left({\text{A}}_{\text{ih}}+{\text{A}}_{\text{hi}}\right)\,\left[{\left({\text{d}}_{\text{jh}}\,\left({\text{N}}_{\text{i}}\right)\right)}^{-1}+{\left({\text{d}}_{\text{hj}}\,\left({\text{N}}_{\text{i}}\right)\right)}^{-1}\right]}{\left({\text{k}}_{\text{i}}^{\text{out}}+{\text{k}}_{\text{i}}^{\text{in}}\right)\,\left({\text{k}}_{\text{i}}^{\text{out}}+{\text{k}}_{\text{i}}^{\text{in}}-1\right)-2\,\sum_{\text{j}\in \text{N}}{\text{A}}_{\text{ij}}\,{\text{A}}_{\text{ij}}})$$

where *d*_*jh*_(*N*_*i*_) is the length of the shortest path between j and h that contains only neighbors of i.

High local efficiency supports parallel processing for the effective integration of information (Bullmore and Sporns, 2009).

#### Measure of Integration

The shortest path length (PL) is the lowest number of edges traveled between any given pair of nodes. The average PL for all node pairs is called the characteristic path length (λ), and it can be mathematically written as $\lambda =\frac{1}{N}\sum_{i\in N}(\sum_{j\in N,j\neq i}\text{PL}/\text{N-1)}$. The average inverse of the shortest path measures global communication efficiency of a network, namely, global efficiency (Latora and Marchiori, 2001), $\text{GE}=\frac{1}{N}\,\sum_{i\in N}\left(\sum_{j\in N,j\neq i}{\left(P\,L\right)}^{-1}/\text{N-1}\right).$ GE represents network integration or overall capacity for parallel information transfer and rapid information exchanges between distributed regions. High GE values indicate high communication efficiency and fewer processing steps between network nodes, and vice versa.

#### Measure of Centrality and Hub Identification

Betweenness centrality (BC) is the fraction of all the shortest paths in the network that pass through the node. Nodes with high BC values act as hubs with many ‘shortest’ paths. Removal of BC nodes considerably changes network performance that is crucial for communication efficiency. We compute this value as follows: ${\text{BC}}_{i}=\frac{1}{\left(N-1\right)\,\left(N-2\right)}\,\sum_{h,j\in N,\,h\neq i,j,i\neq j}\frac{s\,p_{h\,j}\,\left(i\right)}{s\,p_{h\,j}}$, where *sp*_*hj*_ is the number of ‘shortest paths’ between node *h* to node *j*, *sp*_*hj*_(*i*) is the number of shortest paths between node *h* to node *j* that passes through node^®^; BC_*i*_ was computed for all nodes *i* in the network.

We also classified the brain hub of the network according to four nodal parameters: CC, PL, D, and BC. Generally, a hub is characterized by a low CC, short PL, high D, and high BC. Therefore, brain hubs can be identified by determining whether a node fulfilled the score criteria: (1) in the top 20% with lowest CC values; (2) in the top 20% with shortest PLs; (3) in the top 20% with highest Ds; and (4) in the top 20% with highest BC values. Each node was scored between 0–4, as determined by the total number of hub scores. An electrode with a hub score of 2 or higher (HS ≥ 2) was considered a brain hub — the same criteria set by Vandenberghe et al. (2013).

#### Network Sparsity

The directed and weighted brain network was obtained by applying the same statistical threshold to the estimated ECN, but this leads to a slight difference in sparsity. Eventually, the network sparsity was computed by taking the ratio between the total number of existing edges and the maximum number of possible edges in a network [(*N*^2^−*N*)/2 edges].

### Correlation Between Behavioral and Subjective Relevance of Graph Metrics

To assess the relationship between graph metrics of the behavioral and subjective data resulting from color effects, graph metrics were correlated (i) with changes in subjective data (emotional valence and arousal) and (ii) with behavioral data (accuracy and RTs). Firstly, the significant datasets were selected. This was followed by correlation analysis between the changes in subjective data and the brain network metrics by electrodes at group level by computing the Pearson correlation coefficients (r) at *p* < 0.05. The same analysis was repeated for correlation between the behavioral data and the brain network metrics.

### Statistical Analysis

All data are presented as mean (±SD). Since the comparison of mean differences involved three independent groups and more than one dependent variables, one-way multivariate analysis of variance (MANOVA) and Tukey’s HSD *post hoc* test for multiple comparisons (*p* < 0.05) were conducted to determine significant differences between groups. The same statistical analysis was performed for behavioral data and graph metrics separately.

To assess color’s effect on subjective data, a paired *t*-test (*p* < 0.05) was used to identify the significant differences between pre-SAM and post-SAM (ΔV, ΔA, ΔD) at the group level. This was followed by the comparisons between-groups using one-way MANOVA and Tukey’s HSD *post hoc* test.

## Results

In this study, we used EEG to assess the effects of colored MLMs on emotion, cognitive processes during learning and LTM retention. In this section, we present the experiment results in 4 subsections; in terms of the subjective data, the behavioral data, the EEG-based effective connectivity indices and the correlation analysis.

### Subjective Data

**FIGURE 5:**
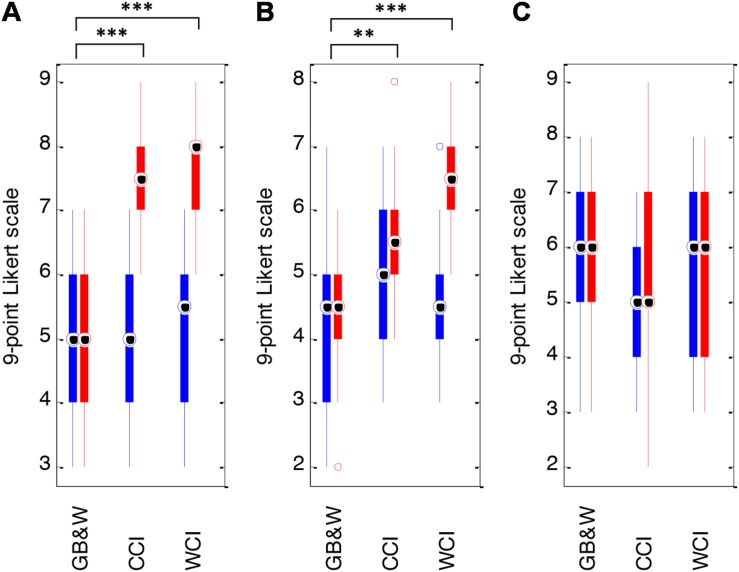
Mean SAM scores: **(A)** valence, **(B)** arousal, and **(C)** dominance for GB&W, CCI, and WCI for an average of 14 participants per condition. Blue boxes = pre-SAM scores and red boxes = post-SAM scores. Asterisks indicate the significance level (^∗∗^*p* < 0.01 and ^∗∗∗^*p* < 0.001).

Color evokes emotional states of valence (pleasure) and arousal. As expected, WCI and CCI groups rated somewhat higher in valence and arousal than the GB&W group. Paired *t*-tests of differences between pre- and post-SAM scores showed significantly differences for valence [*t*(13) = −7.167, *p* < 0.001; from a mean of 5.14 (±1.23) to 7.71 (±0.91)], and for arousal [*t*(13) = −6.853, *p* < 0.001; from 4.64 (±1.15) to 6.43 (±1.02)] in the WCI group. The CCI group showed a significant difference for valence (P) only [*t*(13) = −5.692, *p* < 0.001; from a mean of 5.07 (±1.26) to 7.43 (±1.01)]. No significant differences in valence, arousal and dominance scores were noted for the GB&W group (see Figure 5). One-way MANOVA analysis indicated that a learner’s emotional state was significantly affected by color [*F*(12, 68) = 9.437, *p* < 0.001; Wilk’s Λ = 0.141, ${\text{η}}_{\text{p}}^{2}$ = 0.625]. Colored illustrations had a statistically significant effect on valence [*F*(2,39) = 30.149, *p* < 0.001;${\text{η}}_{\text{p}}^{2}$ = 0.607] and on arousal [*F*(2,39) = 11.813, *p* < 0.01; ${\text{η}}_{\text{p}}^{2}$ = 0.377]. Follow-up MANOVAs with Tukey’s HSD *post hoc* showed significant differences in mean valence scores between CCI and GB&W groups (*p* < 0.001); and between WCI and GB&W groups (*p* < 0.001), but not between CCI and WCI groups (*p* = 0.739). This indicates that participants in CCI and WCI groups experienced a more positive emotional state than the GB&W group. Mean arousal ratings were statistically significant differences between CCI and GB&W groups (*p* < 0.05), and between WCI and GB&W groups (*p* < 0.001), but not between CCI and WCI groups (*p* = 0.125). It is believed that no difference for arousal was observed between the CCI and WCI due to the CCI group being higher in arousal, on the post-SAM task. The side-by-side color-coded boxplots of pre-SAM and post-SAM scores are shown in Figure 5.

### Behavioral Data

**FIGURE 6:**
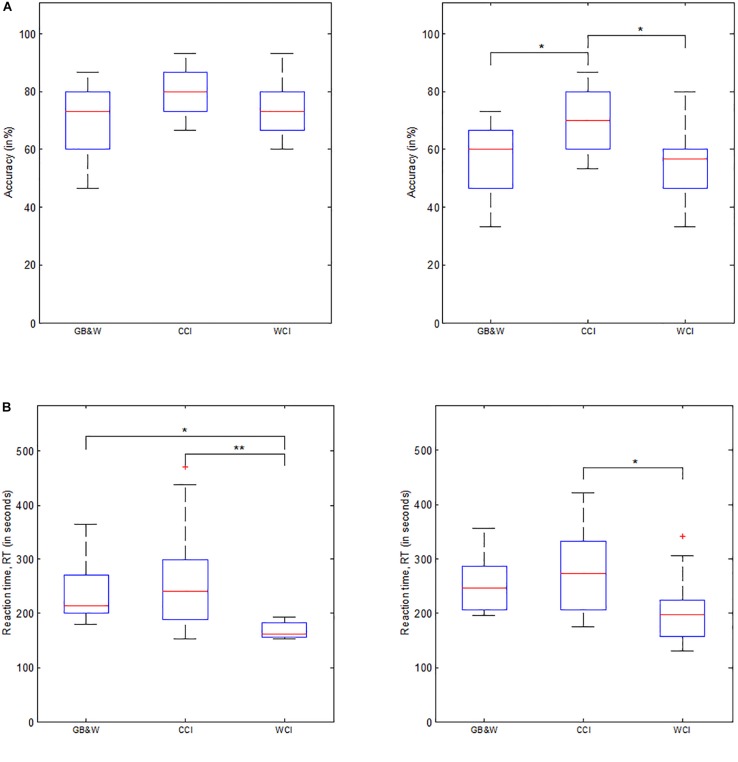
Behavioral data. **(A)** Mean accuracy, and **(B)** Mean reaction time for LTM tests administered after 30-min (left) and 1-month delay (right), respectively. GB&W, CCI, and WCI for an average of 14 participants per condition. Asterisks indicate the significance level (^∗^*p* < 0.05 and ^∗∗^*p* < 0.01).

Behavioral data consists of reaction time (RT) and accuracy (ACC). One-way MANOVA analysis showed that behavioral performance significantly depended on visual color [*F*(8,72) = 3.772, *p* < 0.01; Wilk’s Λ = 0.497, ${\text{η}}_{\text{p}}^{2}$ = 0.295]. Specifically, colored illustrations significantly affect the recall test’s (after 30 min) reaction time, RT_1_ [*F*(2,39) = 8.025, *p* < 0.01; ${\text{η}}_{\text{p}}^{2}$ = 0.292]. However, color had no statistically significant effect on accuracy for the recall test after 30-min, ACC_1_ [*F*(2,39) = 1.853, *p* = 0.17; ${\text{η}}_{\text{p}}^{2}$ = 0.087]. Significant effect on recall test after 1-month, where ACC_2_ results were [*F*(2,39) = 6.111, *p* < 0.001; ${\text{η}}_{\text{p}}^{2}$ = 0.239]; and RT_2_ results were [F(2,39) = 4.369, *p* < 0.05; ${\text{η}}_{\text{p}}^{2}$ = 0.183]. A follow-up analysis by MANOVA with Tukey’s HSD *post hoc* showed mean RT values for recall test after 30-min results were statistically significant differences between GB&W and WCI groups (*p* < 0.05), and between CCI and WCI groups (*p* < 0.01), but not between GB&W and CCI groups (*p* = 0.549). Mean accuracy scores for recall test after 30-min were not significantly different between GB&W and CCI (*p* = 0.167), GB&W and WCI groups (*p* = 0.889), or between CCI and WCI groups (*p* = 0.357). However, delayed memory recall test (after 1 month), mean RT_2_ results were statistically significant differences between CCI and WCI groups (*p* < 0.05); but not between GB&W and CCI (*p* = 0.543) or GB&W and WCI groups (*p* = 0.165). Mean accuracy (ACC_2_) scores were significant differences between GB&W and CCI group (*p* < 0.05), and CCI and WCI groups (*p* < 0.05), but not between GB&W and WCI groups (*p* = 0.994). Overall, results showed higher ACC in both LTM tests (after 30-min and 1-month) are observed for CCI group than WCI and GB&W groups, whereas WCI group responded faster than GB&W and CCI during both LTM tests. Figure 6 illustrates mean scores for accuracy (ACC) and reaction time (RT) for both memory recall tests.

### EEG-Based Effective Connectivity Indices

#### Node Degree

**FIGURE 7:**
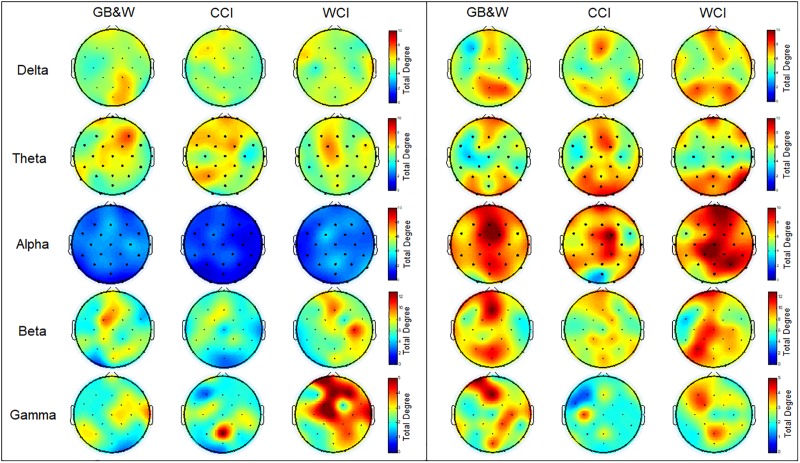
Topological maps of mean total degree (TD): **(left)** resting-state eyes open, and **(right)** learning for all three groups (GB&W, CCI, WCI) and frequencies (delta, theta, alpha, beta, and gamma). The color bars of total degree are set to identical ranges per frequency band. Red colors denote high total degree nodes; Blue colors denote low total degree nodes.

The topographical maps of averaged total degree at each electrode during resting-state eyes open and learning state are shown in Figure 7.

As seen in Figure 7, the total degree of connectivity (TD) increased in most frequency bands (except gamma band) during the learning state compared to resting-state eyes open for all three groups. In the alpha band, the increase of TD observed at mPFC, right PFC, mFC, and bilateral PC, especially for WCI group. In delta and theta bands, higher TD was found for WCI and CCI groups than GB&W group at mPFC and bilateral PC. The beta band showed increased TD in the (i) left PFC, mFC, bilateral PC, mPC and mOC for GB&W group; (ii) mFC, right DLPFC, right FC and PC for CCI group; and (iii) left PFC, mPFC, mFC, bilateral FC and right PC for WCI group. In gamma band, increased TD were observed in the (i) left PFC, mFC, mPC, and mOC for GB&W group; (ii) left PC and mFC for CCI group; and (iii) left DLPFC, left FC and mPC for WCI group.

#### Directionality Index

**FIGURE 8:**
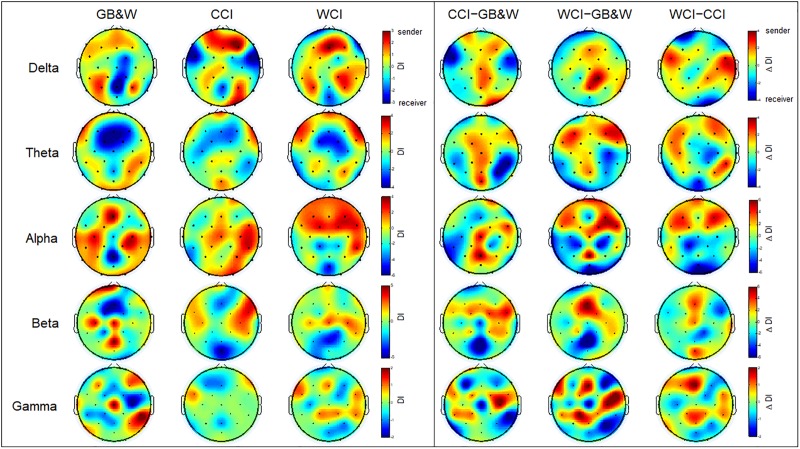
Directionality index (DI). **(left)** Learning condition for all three groups (GB&W, CCI, WCI) and frequencies (delta, theta, alpha, beta, and gamma), and **(right)** Differences between groups for each frequency band. Red colors represent sender nodes; Blue colors represent receiver nodes.

The results of the directionality index (DI) are shown in Figure 8A. In the alpha band, long-range connectivity with information flows from anterior to posterior cortices are observed for WCI group. A similar trend is observed in the theta band, but less sender node is obtained for the CCI group than WCI group. For beta and gamma bands, several observations identified what appeared to be non-systematic activities, particularly, for the beta band. Sender nodes were identified in the (i) left PFC, mFC, left PC, and mPC for GB&W group; (ii) right VLPFC, FC, and PC for CCI group; and (iii) mFC, right FC, and PC for WCI group. In gamma band, sender nodes were appeared at (i) right VLPFC, mFC, and right PC for GB&W group; (ii) right VLPFC and left FC for CCI group; and (iii) mFC, bilateral FC and PC for WCI group. In delta band, similar sender nodes were observed for all groups at bilateral DLPFC and PC. The differences in directionality index between groups were computed (see Figure 8B). Long-range connectivity with information flows from anterior to posterior cortices was observed for WCI compared to GB&W (WCI–GB&W) and CCI (WCI–CCI) in theta and alpha bands. Specifically, in the theta band, sender nodes are observed at bilateral DLPFC, left VLPFC, bilateral FC, and mPC, and that receives at PC, and OC, bilaterally. For the alpha band, sender nodes are observed at bilateral PFC, DLPFC, and FC, and that receives at mOC and bilateral PC.

#### Phase Slope Index: Left vs. Right Hemisphere of Frontal-Parietal-Occipital

In theta band, greater information flows were observed in the left hemisphere (LH) than the right hemisphere (RH) at frontal regions (FP1→FC5) for CCI group. The WCI group showed greater information flows in the RH compared to the LH, specifically at the regions of (1) frontal (FP2→F4 and FP2→FC6), (2) frontal-parietal (FP2→CP6) and (3) parietal-frontal (CP6→FP2). For the GB&W group, information flow in RH is greater than LH at parietal-frontal (CP2→FP2 and CP6→FP2).

In the alpha band, information flow in the RH is greater than LH with long-range synchronization was observed between anterior-posterior regions for both WCI and CCI groups while CCI group had a greater magnitude of information flow in the LH than RH within frontal regions (FP1→FC1 and FC5→FP1). Besides that, a greater magnitude of information flow in the RH than LH within areas of anterior (FC2→FP2) and posterior-anterior regions (CP2→FP2) was observed for the GB&W group.

**FIGURE 9:**
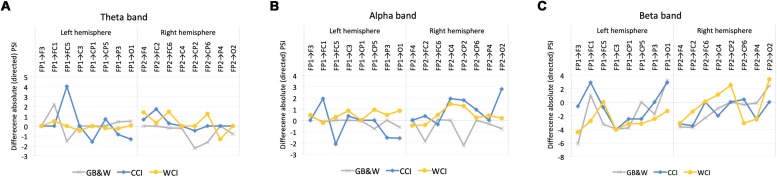
Mean differences in absolute values of PSI within the left (LH) vs. right (RH) hemisphere for **(A)** theta, **(B)** alpha, and **(C)** beta bands, respectively. Positive values: anterior → posterior flows; Negative values: posterior → anterior flows.

In the beta band, greater information flow between frontal-parietal regions (FP2→CP2 and FP2→O2) in the RH while the back-to-front flow was observed in the LH (F3→FP1, C3→FP1, CP5→FP1) for WCI group. For CCI group, a greater information flow in the LH compared to RH (FP1→FC1, FP1→O1, and C3→FP1). Meanwhile, for the GB&W group, a greater magnitude of information flow in the LH (FP1→O1), which followed the back-to-front flow (F3→FP1, C3→FP1, and CP1→FP1). Figure 9 shows the significant differences of mean absolute PSI within hemispheres for electrode pairs.

#### Measure of Segregation

**FIGURE 10:**
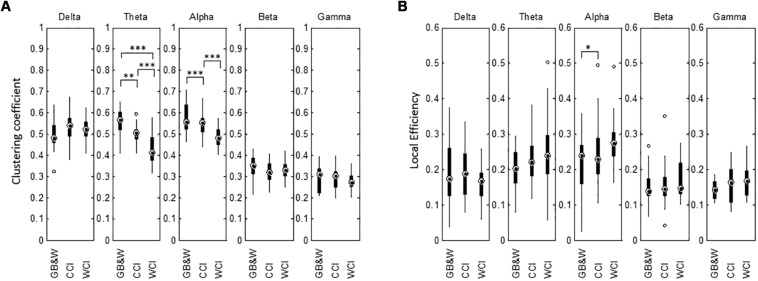
Boxplots show network segregation for **(A)** graph’s clustering coefficient (CC), and **(B)** local efficiency (LE) for all three groups (GB&W, CCI, WCI) and for five frequency bands (delta, theta, alpha, beta, and gamma). Asterisks indicate the significance level (^∗^*p* < 0.05, ^∗∗^*p* < 0.01, and ^∗∗∗^*p* < 0.001).

Results for network segregation using the graph’s clustering coefficient (CC) and local efficiency (LE) are shown in Figure 10. In theta and alpha bands, the CC values obtained show a significant difference between groups. The mean of CC was reduced in theta and alpha bands for WCI group compared to CCI and GB&W groups, while LE increased with decreasing clustering in the following order: [WCI > CCI > GB&W]. In the beta band, CC value for CCI group is lower than WCI and GB&W groups. In the delta band, clustering is increased for the CCI group compared to WCI and GB&W groups. A decrease of clustering with an increase of LE was also found in the gamma but no significant difference was found. Similarly, no statistically significant differences are observed for LE in for all five-frequency bands (except LE in the alpha). Generally, LE is relatively higher for WCI group than CCI and GB&W groups in all bands, except delta, higher LE found in CCI group than WCI and GB&W groups.

#### Measure of Integration

**FIGURE 11:**
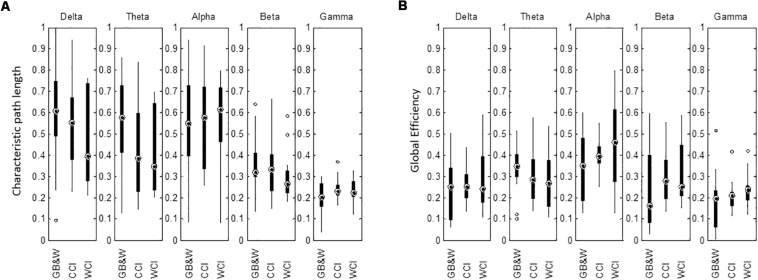
Boxplots show network integration of **(A)** characteristic path length (λ), and **(B)** global efficiency (GE) of the brain network for all groups (GB&W, CCI, WCI) and for five frequency bands (delta, theta, alpha, beta, and gamma).

The mean of characteristic path length (λ) is decreased in the delta, theta, alpha and beta bands for WCI group compared to CCI and GB&W groups. In gamma band, the λ value is slightly higher for the CCI group than WCI and GB&W groups. Besides that, a higher global efficiency (GE) value was observed for the WCI group than CCI and GB&W groups in the delta, alpha, beta, and gamma bands. In theta band, GB&W group showed a higher GE value than CCI and WCI groups. Figure 11 shows boxplots summarizing the results for network integration using λ and GE.

#### Measure of Centrality and Hubs Identification

**FIGURE 12:**
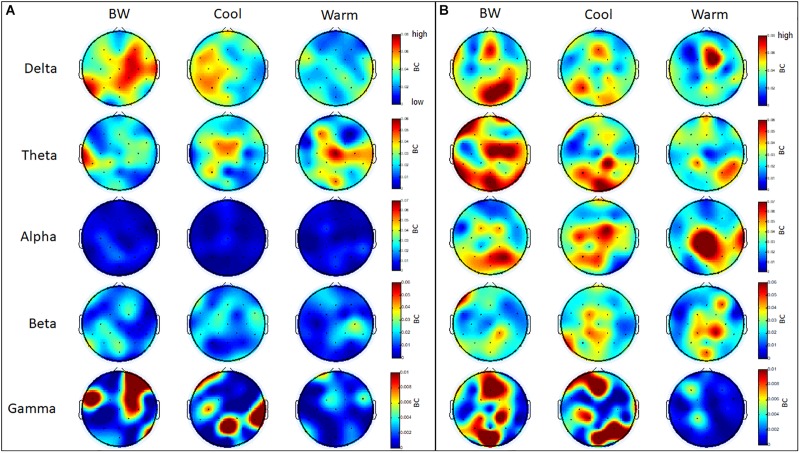
Mean betweenness centrality (BC): **(A)** resting-state eyes open, and **(B)** learning for all three groups (GB&W, CCI, WCI) and for five frequency bands (delta, theta, alpha, beta, and gamma). Red = highest BC value; blue = lowest BC value.

Several regions within frontal, parietal and occipital cortices have a higher level of betweenness centrality (BC) in the theta and gamma bands for GB&W group than CCI and WCI groups. In delta band, all three groups exhibited higher levels of BC values at mPFC, whereas a slight increase of BC values at mOC and right PC is observed for the GB&W group. In the alpha band, several areas within the mFC, PC and right TC have a higher level of BC than other regions for WCI group compared to CCI and GB&W groups. In gamma band, a slight increase of BC values at right VLPFC, mFC, right PC and mOC were observed for the GB&W group compared to CCI and WCI groups. Lowest BC nodes were observed for WCI group. Figure 12 presents the betweenness centrality (BC) values obtained at each electrode.

Besides, brain hubs of the network were also identified using hub score, HS (see Section Measure of Centrality and Hub Identification). Results showed that more nodes met the HS criteria were revealed for the CCI group in theta and alpha bands than WCI and GB&W groups. It can be seen in the Supplementary Tables S1–S3, seven brain hubs were identified in the CCI group in the theta band, which is followed by six brain hubs in GB&W group and four brain hubs in WCI group. In the alpha band, the sequence as follows: CCI (8 brain hubs) > WCI (7 brain hubs) > GB&W (6 brain hubs). In the beta band, more nodes met the HS criteria was found in the WCI than that of CCI and GB&W groups [WCI (10 brain hubs) > CCI = GB&W (7 brain hubs)].

#### Network Sparsity

There was no significant difference in mean sparsity of the brain network between-groups during EO condition across the five frequency bands, *F*(10,70) = 0.799, *p* = 0.630; Wilk’s Λ = 0.806, ${\text{η}}_{\text{p}}^{2}$ = 0.102; and during L condition, *F*(10,70) = 0.240, *p* = 0.991; Wilk’s Λ = 0.935,${\text{η}}_{\text{p}}^{2}$ = 0.033, as determined by one-way MANOVA, and thus no further follow-up tests were performed as it has not achieved a statistically significant result. This showed that the variations in sparsity impart a negligible effect on the topological organization of the resulting brain networks and the comparison of network metrics between groups is unbiased. Furthermore, the values of network sparsity within the range of 0.05–0.5 (≡ 5–50%), indicating the complex network of human brain (Achard and Bullmore, 2007; Bullmore and Sporns, 2012). The mean sparsity of the brain networks of each frequency band for EO and L conditions are summarized in Table 1.

**TABLE 1:** Mean sparsity of the brain networks during eyes open (EO) and learning (L) conditions.

**Frequency band**	**Group**	**EO condition**		**L condition**	
		**Mean**	**Standard Deviation**	**Mean**	**Standard Deviation**
Delta	BW	0.229	0.061	0.196	0.065
	CCI	0.184	0.087	0.198	0.090
	WCI	0.197	0.104	0.222	0.128
	Total	0.203	0.086	0.205	0.096
Theta	BW	0.197	0.088	0.235	0.054
	CCI	0.212	0.139	0.221	0.129
	WCI	0.196	0.085	0.221	0.073
	Total	0.202	0.104	0.226	0.089
Alpha	BW	0.078	0.049	0.274	0.142
	CCI	0.052	0.031	0.256	0.151
	WCI	0.080	0.041	0.287	0.120
	Total	0.070	0.042	0.272	0.135
Beta	BW	0.221	0.109	0.300	0.181
	CCI	0.186	0.066	0.274	0.148
	WCI	0.241	0.145	0.303	0.145
	Total	0.216	0.111	0.292	0.156
Gamma	BW	0.083	0.078	0.101	0.089
	CCI	0.077	0.064	0.074	0.048
	WCI	0.126	0.128	0.094	0.048
	Total	0.095	0.094	0.090	0.064

### Correlation Analysis Between Graph Metrics and Behavioral/Subjective Measures

We correlated graph metrics with changes in emotional valence (ΔV) and arousal (ΔA), and behavioral performances (RT and Accuracy). Specifically, CC and BC values for theta and alpha bands; while local efficiency (LE) for the alpha band were selected, because only these bands showed significant differences between groups. On the other hand, the significant change in valence for CCI and WCI groups, arousal for WCI group, reaction time for WCI and accuracy for CCI were selected (represented as ΔV_CCI_, ΔV_WCI_, ΔA_WCI_, RT_1,*WCI*_, ACC_2,*CCI*_). Subscripts 1 and 2 denote memory recall after 30-min and 1-month delay, respectively.

For ΔV, a significant negative correlation between BC and ΔV_CCI_ in the mPC was obtained in the theta band. For the alpha band, CCI and WCI groups obtained opposing results: CC was positively correlated with ΔV_CCI_ in the right PC, but negatively correlated with ΔV_WCI_ in the left VLPFC, left TC, right PC, and mOC. BC and LE relationships with ΔV in the alpha band for WCI and CCI groups revealed similar results, with BC positively correlated with ΔV_CCI_ in the mFC (but negatively with right FC), and with ΔV_WCI_ in the right FC. LE was positively correlated with ΔV_CCI_ in the mPFC and left TC, and with ΔV_WCI_ in the left PFC. For ΔA, significant negative correlations were found between CC in the theta and alpha bands with ΔA_WCI_ in left DLPFC and TC and right PC. BC was negatively correlated with ΔA_WCI_ in the left PFC but positively correlated with ΔA_WCI_ in the right PC in the theta band. BC was positively correlated with ΔA_WCI_ in the right PC together with a positive correlation between LE and ΔA_WCI_ in the left OC results in the alpha band.

Behaviorally, correlations were positive for both CC and BC in the theta band for ACC_2,*CCI*_ in the left FC and right DLPFC, respectively. But negative for both CC and BC in the alpha band for ACC_2,*CCI*_. Specifically, CC is negatively correlated with ACC_2,*CCI*_ in the right PC; BC is negatively correlated with ACC_2,*CCI*_ in the left FC and TC. As for RT, a negative relationship between CC and RT_1,*WCI*_ was observed for the theta band in the mPFC; BC is positively correlated with RT_1,*WCI*_ in the left FC, bilateral PC and mPC in the alpha band. The results of the correlation analysis are summarized in Table 2.

**TABLE 2:** Correlation analysis (significant findings only) between graph metrics & SAM (**ΔV_CCI_**, **ΔV_WCI_**, **ΔA_WCI_**) and behavioral tests (**RT_1,*WCI*_**, ACC_2,*CCI*_).

**EEG bands**	**Graph metrics**	**ΔV_CCI_**	**ΔV_WCI_**	**ΔA_WCI_**	**ACC_2,*CCI*_**	**RT_1,*WCI*_**
Theta	CC	–	–	F3	FC5	FPz
				(−0.73, 0.003)	(0.56, 0.044)	(−0.55, 0.043)
				T7		
				(−0.62, 0.018)		
				P8		
				(−0.57, 0.033)		
	BC	Pz	–	FP1	F4	–
		(−0.71, 0.004)		(−0.55, 0.042)	(0.54, 0.044)	
				CP2		
				(0.544, 0.044)		
Alpha	CC	P4	F7	FPz	CP6	–
		(0.60, 0.025)	(−0.71, 0.005)	(−0.60, 0.024)	(−0.55, 0.042)	
			T7	F4		
			(−0.54, 0.047)	(−0.59, 0.027)		
			P4	FC6		
			(−0.55, 0.042)	(−0.64, 0.015)		
			POz	P4		
			(−0.56, 0.039)	(−0.69, 0.006)		
				P8 (−0.54, 0.048)		
	BC	Fz	FC6	P4	FC1	FC1
		(0.54, 0.047)	(0.63, 0.017)	(0.67, 0.009)	(−0.55, 0.040)	(0.62, 0.018)
		FC2			T7	CP5
		(−0.64, 0.013)			(−0.59, 0.027)	(0.56, 0.039)
						CP2 (0.68, 0.008)
						Pz (0.54, 0.046)
	LE	FPz	FP1	O1	–	–
		(0.67, 0.013)	(0.55, 0.040)	(0.55, 0.041)		
		T7				
		(0.59, 0.033)				

*Numerical inside bracket indicates (*r, p*) where *r* is the Pearson correlation coefficients and *p* is the *p-*values.*

## Discussion

The experiment results showed that colored MLMs could induce a positive emotional state on the learner, activate the brain to focus and process information, and that improve learning (higher accuracy). The following of this section discusses several findings observed from experiments. Firstly, we noticed a non-identical magnitude of information flow within the left and right hemispheres, by which the information flows are dependent on the use of color in MLM design. Secondly, the directionality indices showed the long-range frontal-parietal connectivity in theta and alpha bands, and it leads to top–down processing for WCI and CCI groups, whereas, bottom–up processing is observed for GB&W group due to the reverse information flow obtained from parietal to frontal. These findings suggested the top-down modulation of working memory stimulated by color, which also appeared to enhance anticipatory control over encoding and retention (Zanto et al., 2010). Specifically, the PFC region appears to exert top-down attentional control while also influencing information selection in working memory for encoding; thus, engaging cognitive control while learning (Muhle-Karbe et al., 2017). Furthermore, selective attention facilitates the integration of sensory and perceptual information to be stored in different brain areas (Chai et al., 2017). This suggests that long-range connectivity between frontal and multimodal association areas (theta-alpha range, 4–13Hz) is likely associated with active monitoring and enhanced performance (Zanto et al., 2011). Studies have reported that color processing involves brain regions of association areas, visual, frontal and prefrontal (DLPFC and VLPFC) cortices (Zeki and Marini, 1998; Vandenbroucke et al., 2014). Overall, the measure of the emotional and cognitive process during learning using EEG in this study has quantitatively proved that the brain has a higher response to the colored MLMs than achromatic MLMs.

### Subjective Data

Participants who were exposed to WCI materials rated somewhat higher emotional valence (more enjoyable) and arousal (more excited/activated) than CCI and GB&W groups, whereas CCI group reported high emotional valence (more pleasant and relaxed) but with less arousal (calm). According to color psychology, the feeling of people is stimulated by warm colors whereas cool colors induce a sense of relaxation and calming effect (Elliot and Maier, 2014). Besides that, color heightened motivation on the learner to continue learning from the materials as the color might be perceived as more vivid and interesting (Navratil et al., 2018). Our experiment shows that colored MLMs are able to induce positive emotion on participants while a similar finding was reported by Um et al. (2007), Um et al. (2012), Mayer and Estrella (2014), Le et al. (2018), Münchow and Bannert (2018), Navratil et al. (2018), and Uzun and Yıldırım (2018); which confirmed the effect of color on emotional experiences. Positive emotion strengthens motivation, attention and behavioral intentions that facilitate learning and enhance memory (Pekrun, 1992) as hypothesized by the Broaden-and-Build theory (Fredrickson, 1998). In addition, psychophysiological and neuroscience studies of emotional processing reported that pleasant states are associated with the brain’s appetitive motivational (SEEKING) system that might influence the degree of attentive engagement during perceptual processing (Panksepp, 2005; Lang and Bradley, 2010).

### Behavioral Data

Behaviorally, the CCI group had a higher accuracy rate than GB&W and WCI groups, although no significant mean differences of ACC_1_ were obtained. However, the 1-month recall revealed a significant increase of mean accuracy (ACC_2_) for the CCI group compared to WCI and GB&W groups. This indicates that color induced a more substantial influence on 1-month recall (longer LTM retention) than that for 30-min recall. This demonstrates that color has an effect on delayed LTM instead of immediate LTM, which improves long-term retention of learned information, presumably by increasing ones’ attention, emotional valence and arousal (Cano et al., 2009; Dzulkifli and Mustafar, 2013).

In terms of reaction time, WCI group respond faster than GB&W and CCI groups during the 30-min recall, and with a significant difference between CCI and WCI groups. This might be due to the fact that warm colors are more stimulating, leading to faster responses (attention and arousal ↑, RT↓), whereas, cool colors are more relaxing and calming that maintains focus and concentration during learning (concentration ↑, accuracy ↑). This suggests that warm colors could induce higher levels of arousal, excitement, and attention to the learner (Wexner, 1954) than the cool colors. However, the levels achieved cannot be too high that could cause a performance decrease according to the Yerkes-Dodson Law. Compared to warm colors, cool colors elicit more significant feelings of relaxation and calmness (Wexner, 1954), where learners will be able to keep focused and concentrated while learning, and will facilitate memory consolidation for LTM retention (Nava et al., 2004).

### Node Degree

As referred to Figure 7 in Section “Node Degree,” an increased total degree was observed, indicating that brain regions are organized and integrated globally when exposed to colored MLMs compared to achromatic MLMs. The result showed that high degree nodes at prefrontal, frontal and posterior association cortices (temporal, parietal and occipital) in beta and lower-frequency (0.5-13 Hz) bands. This finding suggests that memory improvement with longer knowledge retention can be achieved by increasing the connectivity between frontal and posterior areas, as it was supported by Bassett and Mattar (2017). This is explained by the dominant role of prefrontal and frontal cortices during the processing of visuospatial information in working memory (Johnson et al., 2018), which prioritizes sensory input during learning (Muhle-Karbe et al., 2017) associated with cognitive-emotional functions (Tyng et al., 2017) that facilitate successful encoding and retrieval — with the presence of color. Besides, multimodal association cortices are referred to as higher-order association areas, including (i) the prefrontal areas that have been linked with working memory as well as with attentional, emotional and motivational processing and color processing; and (ii) posterior association areas of temporal and parietal are linked with declarative (semantic and conceptual) knowledge and experiences and occipital is responsible for visual information processing are crucial for learning and memory (Mesulam, 1998). These support the idea of the color evokes emotional experiences and modulates global connectivity (Kinnison et al., 2012).

In the gamma band, high degree nodes were mostly found in prefrontal and frontal cortices for GB&W group compared to CCI group, where high degree regions were found in left PFC, mFC, and mPC. This is likely due to increased working memory (WM) load as WM load-related gamma band network identified at prefrontal and parietal areas (Roux et al., 2012). For CCI and WCI groups, it is apparent that the high degree nodes increased at lower extent, which could be explained by the decreased working memory load due to cognitive control of engagement (Muhle-Karbe et al., 2017) resulting from the color effect that mediate the manipulation of the contents of working memory to be remembered as increased degree found at left DLPFC (Curtis and D’Esposito, 2003). These findings substantiate the importance of color in learning that might contribute to efficient information processing (Wu et al., 2017).

### Directionality Index

These results can be seen in Figure 8 (see Section Directionality Index). Long-range anterior-posterior connectivity from prefrontal and frontal regions to posterior cortical regions (theta and alpha bands) suggest stronger interactions between the executive network (DLPFC) and higher-order association areas, elicited by warm colors for WCI group. The finding of increased anterior-posterior connectivity in the theta band was more significant for the WCI group but not observed in the alpha band for CCI and GB&W groups. These results suggest that color strongly influences the direction of information flow, which is consistent with top–down processing by prefrontal and frontal cortices to parietal, temporal and occipital regions when focused on external stimuli for optimized learning. This finding also agrees with reports by others (Zanto et al., 2011; Muhle-Karbe et al., 2017) and is indirectly linked with top–down attentional processes in theta and alpha activity, due to emotional responses to color in DLPFC and VLPFC cortices that associated with working memory and emotional regulation processes (Buhle et al., 2014). Moreover, a review (Klimesch, 2012) also reported that theta and alpha are associated with top-down control processes in two large storage systems (working memory and long-term memory). Based on results obtained, it supports that the blue color increases DLPFC and VLPFC activation, which eventually improved the working memory performance. The similar finding was reported by Alkozei et al. (2016), who stated that blue light could induce a higher activation of DLPFC and VLPFC than amber control light. Thus, it is suggested that the measurement of EEG can be used to assess the influence of colored MLMs on LTM retention and learning.

### Left vs. Right Hemisphere of Phase Slope Index and Color

The differences in the magnitude of information flows are depicted in Figure 9, with right hemisphere (RH) predominance, particularly for warm colors in anterior regions and anterior-posterior flows for theta and beta bands with greater RH flows from anterior-posterior regions in the alpha band. Cool colors produced greater information flow in LH than RH at anterior regions in theta and beta bands, whereas greater anterior-posterior flows in RH in the alpha band. Larger anterior to posterior flows are likely a valence-related effect from color via top–down modulation of the prefrontal cortex and parietal regions that enhance semantic processing, as well as working memory and attentional processes as reported by Dolcos and Denkova (2014). Greater information flows within anterior regions in the left hemisphere for cool colors, likely representing attention and positive emotional valence, as reported by Aftanas and Golocheikine (2001), where alertness enhanced cognitive performance (Alkozei et al., 2016). A different pattern was observed for the GB&W group which greater information flows in RH from posterior to anterior regions in theta and alpha bands. This indicates that achromatic MLMs produced emotional distractions that required bottom–up processing (parietal to prefrontal cortex) to avoid unfavorable outcomes (Dolcos and Denkova, 2014).

### Measure of Segregation

Network segregation was characterized by the clustering coefficient and local efficiency (see Figure 10). Significantly reduced clustering was observed in theta and alpha bands for WCI group compared to CCI and GB&W groups. This possibly favors the global integration of information exchanges (greater long-range connectivity) between different brain areas. Decreased clustering coefficient in the alpha network also supports greater cognitive effort on working memory to optimize information processing suggested by Kitzbichler et al. (2011) as well as the encoding, storage, and retrieval of stored memories (Toppi et al., 2018). These findings show a decline in functional segregation that was associated with increased local efficiency (global network integrity) between brain regions, i.e., with increased long-range anterior-posterior connectivity (higher magnitude of information flows). High local efficiency indicates a high fault tolerance to maintain effective communication. When greater cognitive effort, brain network becomes less clustered, and more long-range synchronization provides “short-cuts” between cortical areas in alpha, beta and gamma network (Kitzbichler et al., 2011). However, in our case, we found a less clustered brain network in the theta, alpha, beta and gamma bands for WCI and CCI groups compared to GB&W. This could be because color evoked emotion that emotional and motivational processing enhanced long-range connectivity as previously reported (Aftanas and Golocheikine, 2001; Kinnison et al., 2012).

### Measure of Integration

Results of network integration are shown in Figure 11. There were no significant differences: neither decrease in characteristic path length (λ) nor increase in global efficiency (GE) for all three groups and all five EEG sub-bands, making it apparent that color did not influence network integration. The λ decreases with increase in GE for WCI and CCI groups in the delta, theta, alpha and beta bands could be attributed to optimal information processing globally (Toppi et al., 2018) due to fewer processing steps as discussed in Section “Graph Theoretical Analysis: Measure of Integration” and increased active maintenance of working memory. As discussed earlier (Roux and Uhlhaas, 2014), when one’s experience is more positively valenced (Koelstra et al., 2012), the brain appears to enhance anticipation and thus controls attention in support of stronger encoding and maintenance of visual working memory (van Driel et al., 2017; Toppi et al., 2018). Our results agreed with the use of color as an emotional design element to channel instructional content and optimize learning as observed by Um et al. (2012), Mayer and Estrella (2014), Plass et al. (2014), Le et al. (2018), Stark et al. (2018), Uzun and Yıldırım (2018). However, further work is required to assess the color’s non-significant effect on network integration.

### Measure of Centrality and Hubs Identification

Examination of betweenness centrality (BC) showed multimodal association areas in prefrontal, posterior parietal and occipital regions, with significantly higher levels of BC in theta and beta bands for GB&W and CCI groups, compared to WCI. We believe this is due to an increased working memory load (achromatic and cool colors), which increases BC for more efficient communication and for integrating different cognitive processes (Dai et al., 2017). WCI group had the lowest number of BC nodes, primarily involving the medial frontal (Cz) in the alpha band, but with some nodal concentrations in temporal and posterior parietal lobes. A decrease in frontal BC nodes suggests these nodes did not accelerate information exchange (WM demand ↓) for warm colors. However, increased BC over temporal and posterior regions indicate participation in information exchange acceleration between memory and association networks in support of successful encoding and retrieval (Dai et al., 2017).

Moreover, a greater number of hubs coincided with a higher level of BC nodes, only for CCI group, potentially accessing a higher accuracy rate—specifically, left VLPFC (F7), left parietal (P3), right temporal (P8) and occipital regions (POz and O2) in theta networks. Although alpha band evinced more brain hubs for the CCI group, with fewer nodes being pivotal nodes (FC2, Cz and P3) were observed, compared to WCI and GB&W groups. Thus, our analyses support the suggestion that successful memory retrieval involves greater overall connectivity across multiple cortical areas in the theta band. Nonetheless, ECN appeared to be marked by specific brain hubs evoked by color (Schedlbauer et al., 2014). Perceived color produces different sensations because of light reflectance. Visual pathways begin at the retina and extend to occipital regions, the fusiform gyrus, as well as to the inferior temporal, parietal and prefrontal cortices (Zeki and Marini, 1998). Color recruits temporal and parietal cortices plus the dorsal attention network could improve learning and increase retention of learned information, as observed in the CCI group’s accuracy for both 30-min recall and 1-month recall, compared to the WCI and GB&W groups. Moreover, in the beta band, more brain hubs are found regions, including right PFC, mFC, right DLPFC, bilaterally PF and left OC when subjects learned from WCI materials. This could be explained by warm colors that activate the brain and, thus, more connections and processing of information and that leads to faster responses during recall tests.

### Correlation Analysis Between Graph Metrics and Behavioral/Subjective Measures

Colored content had the most considerable influence on theta and alpha bands, suggesting that these bands correlate with emotional valence, motivation, and attention that can be used as psychophysiological indicators to assess changes in emotional responses to learning materials (Aftanas and Golocheikine, 2001; Koelstra et al., 2012; Shu et al., 2018). Results in Table 2 show that color effects were reflected in graph metrics from ECN. Warm colors increased emotional valence in association with reduced local CC, increased BC and LE in the alpha band, found at most regions of the frontal (PFC, VLPFC, and FC), temporal and posterior association areas, suggesting that alpha networks favor global integration over local segregation for efficient information processing.

In terms of emotional arousal, increased arousal levels were negatively correlated with reduced local CC in theta and alpha bands and theta betweenness centrality, but with increased betweenness centrality and local efficiency in the alpha band—mostly in frontal and posterior parietal regions. This suggests higher arousal. More interactions between frontal and posterior parietal regions suggest that color initiates important top-down processing. Behaviorally, increased accuracy was linked to increased CC and betweenness centrality in the theta band; but with decreased local CC and betweenness centrality in the alpha band. Also, reduced reaction times correlated with reduced BC in alpha band but with increased CC in the theta band. Overall, declines in local CC values indicate increased memory demand, as reported by Toppi et al. (2018). Thus, EEG-based connectivity is key to describing the relationship between users and technological tool designers. It highlights emotional processes in response to color effects on memory encoding and retrieval.

## Limitations and Future Directions

We provided evidence that color is associated with brain connectivity by estimating ECN and by characterizing patterns of ECN with graph metrics. Nonetheless, several limitations indicate a need for further study. First, the current study has only 45 subjects recruited; more subjects need to be recruited in order to achieve generalization of the study. Second, using only three-color schemes for experimental manipulation does not reflect real-world technological tools that display a wide range of color. More neuroimaging-based studies are needed for better understanding of the color effects on brain connectivity, emotional experiences and behavioral responses using more color combinations. Third, color hue alone is insufficient for encoding applications where serious consequences can result when color-deficient users make incorrect selections. Our analysis covered brain interregional interactions at the sensor-level with the use of a robust measure of effective connectivity and surface Laplacian. It is important to note that the surface Laplacian approach as adopted in this study mitigated the volume conduction problem without addressing it completely as mentioned in Section “Surface Laplacian Transformation.” The actual measurement of brain connectivity could only be achieved at the source-level. This would require high-density EEG recording (min. electrodes ≥ 64) along with simultaneous fMRI scanning (He et al., 2019). In addition, it is also important to compare the results of the whole pipeline of source reconstruction and connectivity estimation by using different combinations of forward and inverse models, and connectivity measures due to limitation of — (1) source reconstruction caused by residual signal leakage at source level (Palva et al., 2018), and (2) estimation of connectivity caused by source mixing (Mahjoory et al., 2017).

## Conclusion

This paper reports the first investigation about effective connectivity network (ECN) under different color-related learning conditions. The work proposed the use of directional connectivity and characterized network topologies (by graph theory analysis) to assess the effect of color on emotional experiences and memory performance. This study demonstrated that colored multimedia learning materials induced positive emotional experiences during learning and influenced the brain’s information processing, as reflected by ECN based on EEG signals. The positive emotion increased motivation to learn with anticipatory, top–down information processing found in the theta and alpha bands which leads to improved LTM retention and recall. Overall, combining subjective and behavioral findings, we believe that EEG observations on the influence of color on emotion and cognitive process during learning could serve as a foundation that improves the design of learning materials.

## Data Availability Statement

The datasets generated for this study are available on request to the corresponding author.

## Ethics Statement

Experimental protocols were approved by the Medical Research Ethics Committee (MREC) of the Royal College of Medicine Perak, Universiti Kuala Lumpur (RCMP UniKL). Informed consent was obtained from all participants.

## Author Contributions

MC, AM, and MA contributed to the experimental design, data processing, and data analysis. MC also participated in data acquisition, drafted and revised the manuscript. HA, LI, and MS contributed to the data processing and data analysis. TT contributed to the data processing, data analysis, and revised the manuscript. All authors read and approved the final manuscript.

## Conflict of Interest

The authors declare that the research was conducted in the absence of any commercial or financial relationships that could be construed as a potential conflict of interest.
