# Supplementary material for: Exploring EEG Effective Connectivity Network in Estimating Influence of Color on Emotion and Memory
Source: Front Neuroinform. 2019 Oct 9;13:66. doi: 10.3389/fninf.2019.00066 (PMC6794354; doi:10.3389/fninf.2019.00066)
Supplement: Supplementary file 1 [file Table_1.DOCX]

## Power Spectral Density (PSD) Analysis

EEG power spectra for EO and L conditions were computed using Welch’s method ([Welch, 1967](#_ENREF_14)) with a Hanning window length of 4096 and 50% overlap (for lower frequency bands <13Hz) thus yielding a 0.5 Hz frequency resolution. Then, a Hanning window length of 2048 and 50% overlap and resulting in 1 Hz frequency resolution were used for higher frequency bands (>13Hz). The absolute power was retained on its own values for comparison of the power spectra between group differences in response to task conditions, and thus no log-transformation was performed. The mean power of the frequency bands of interest were computed for Delta (0.5−4 Hz), Theta (4−8 Hz), Alpha (8−13 Hz), Beta (13−30 Hz), and Gamma (30−48 Hz) by averaging power values across frequency bins. The differences between groups of the mean power values in each electrode for each of the frequency bands were determined by Kruskal-Wallis test. Post-hoc tests by means of Mann-Whitney U tests were carried out on 3 different combinations of groups (^3^C_2_) to examine the actual differences. Bonferroni adjustment was made for multiple comparisons to minimize the risk of type I errors, and that resulted in a new significance level of 0.05/3 = 0.017 (*p* < 0.017).

There were no significant differences in mean PSD between groups for all frequency bands (except beta at left PFC). PSD results showed increased delta at prefrontal cortex (PFC) and ventrolateral prefrontal cortex (VLPFC) while it decreases at posterior regions were observed for all three groups. This could be due to continuous-attention task of learning as delta has associated with internal attention ([Harmony, 2013](#_ENREF_3)). Besides, increased theta power was observed at prefrontal, frontal and occipital areas for all three groups. This is presumably due to theta band is involved in active working memory maintenance and recall of stored information ([Jensen & Tesche, 2002](#_ENREF_5)). The increment of theta power at frontal midline observed in CCI group might due to pleasantness and is also related to attentional process ([Bush, Luu, & Posner, 2000](#_ENREF_2); [Sammler, Grigutsch, Fritz, & Koelsch, 2007](#_ENREF_11)).

Alpha oscillation is inhibitory rhythm and thus a reduction of alpha band was found in all groups. However, alpha oscillation still can be found at prefrontal and posterior regions, which indicating that attention and memory processes ([Klimesch, 1999](#_ENREF_7)) that related to top-down processing ([Von Stein, Chiang, & König, 2000](#_ENREF_13)). The power of beta band was found to be increased at bilaterally frontal cortex and temporal regions in the WCI and CCI groups compared to GB&W, indicating alertness, concentration, motivation and enhanced memory formation ([Rowland, Meile, & Nicolaidis, 1985](#_ENREF_10); [Scholz, Schneider, & Rose, 2017](#_ENREF_12)), which agreed with previous reports on emotion and working memory ([Bunzeck, Guitart-Masip, Dolan, & Düzel, 2011](#_ENREF_1); [Scholz et al., 2017](#_ENREF_12)) associated with greater recall. Study ([Kortelainen, Väyrynen, & Seppänen, 2015](#_ENREF_8)) also showed that emotional arousal (excited/activated) can be predicted by the increased beta power at frontal regions. Increased beta power observed at posterior temporal regions, bilaterally in the GB&W could be linked cognitive processing ([Ray & Cole, 1985](#_ENREF_9)). Increased gamma power found at frontal and parietal cortex, bilaterally might be related to acquisition and retrieval of memory ([Klimesch, 1996](#_ENREF_6)). Meanwhile, increased gamma power found at prefrontal cortex in GB&W might due to increase working memory load ([Howard et al., 2003](#_ENREF_4)). Topographical maps of the averaged PSD for each frequency band are presented in Figure 1. Units for all plots are ($({{\mu V}/{\mathrm{cm}^{2}})}^{2})$.

Figure S1. Topographical maps of the differences in mean PSD between L and EO for five frequency bands: delta, theta, alpha, beta and gamma, respectively. GB&W (left column), CCI (middle column), and WCI (right column). Red color indicates increase power in L compared to EO; blue color indicates decrease power in L compared to EO.

Supplementary Tables

Table S1. Theta band. Hub identification according to the four graph metrics of the degree (D), path length (PL), clustering coefficient (CC) and betweenness centrality (BC);
 for each node and group: GB&W (left), CCI (middle), WCI (right), respectively. — symbol † denotes BC nodes. Bold rows are represented the identified brain hubs with hub scores, HS ≥ 2. Overall, in the theta band, CCI group showed higher number of hubs compared to GB&W and WCI groups.

| **GB&W (Group 1)** | | | | | |  | **CCI (Group 2)** | | | | | |  | **WCI (Group 3)** | | | | | | |
| --- | --- | --- | --- | --- | --- | --- | --- | --- | --- | --- | --- | --- | --- | --- | --- | --- | --- | --- | --- | --- |
| **Nodes** | **D** | **PL** | **CC** | **BC** | **HS** |  | **Nodes** | **D** | **PL** | **CC** | **BC** | **HS** |  | **Nodes** | **D** | **PL** | **CC** | **BC** | **HS** |  |
| FP1 | 7 | 0.45 | 0.25 | 0.052 | 0 |  | FP1 | 7 | 0.34 | 0.28 | 0.038 | 0 |  | FP1 | 7 | 0.30 | 0.37 | 0.018 | 0 |  |
| **FPz** | **8** | **0.27** | **0.32** | 0.039 | **2** |  | FPz | 7 | 0.30 | 0.20 | 0.038 | 1 |  | **FPz** | **9** | **0.28** | **0.37** | 0.045**^†^** | **2** |  |
| **FP2** | **8** | **0.50** | **0.53** | **0.060^†^** | **2** |  | FP2 | 6 | 0.24 | 0.33 | 0.018 | 0 |  | FP2 | 6 | 0.34 | 0.41 | 0.010 | 0 |  |
| **F7** | **7** | **0.41** | **0.23** | **0.075^†^** | **2** |  | **F7** | **6** | **0.35** | **0.22** | **0.048^†^** | **2** |  | F7 | 7 | 0.35 | 0.20 | 0.035 | 1 |  |
| **F3** | **4** | **0.24** | **0.23** | **0.042** | **2** |  | F3 | 5 | 0.37 | 0.28 | 0.013 | 0 |  | F3 | 5 | 0.29 | 0.39 | 0.030 | 0 |  |
| Fz | 7 | 0.37 | 0.36 | 0.030 | 1 |  | Fz | 8 | 0.29 | 0.26 | 0.034 | 1 |  | Fz | 6 | 0.19 | 0.34 | 0.035 | 1 |  |
| F4 | 5 | 0.34 | 0.39 | 0.016 | 0 |  | F4 | 6 | 0.24 | 0.27 | 0.036 | 0 |  | F4 | 6 | 0.24 | 0.34 | 0.021 | 0 |  |
| F8 | 5 | 0.30 | 0.32 | 0.022 | 0 |  | F8 | 5 | 0.27 | 0.12 | 0.023 | 1 |  | **F8** | **8** | **0.25** | **0.24** | 0.036**^†^** | **3** |  |
| FC5 | 4 | 0.39 | 0.16 | 0.014 | 1 |  | **FC5** | **4** | **0.18** | **0.20** | **0.009** | **2** |  | FC5 | 6 | 0.21 | 0.27 | 0.031 | 1 |  |
| FC1 | 6 | 0.22 | 0.43 | 0.040 | 1 |  | FC1 | 6 | 0.22 | 0.26 | 0.029 | 1 |  | FC1 | 5 | 0.18 | 0.35 | 0.029 | 1 |  |
| FC2 | 5 | 0.29 | 0.46 | 0.051 | 1 |  | **FC2** | **8** | **0.16** | **0.35** | **0.035** | **2** |  | FC2 | 5 | 0.14 | 0.30 | 0.026 | 1 |  |
| FC6 | 6 | 0.54 | 0.42 | 0.045 | 0 |  | FC6 | 5 | 0.30 | 0.29 | 0.018 | 0 |  | FC6 | 6 | 0.34 | 0.49 | 0.020 | 0 |  |
| T7 | 6 | 0.41 | 0.37 | 0.045 | 0 |  | T7 | 6 | 0.31 | 0.40 | 0.022 | 0 |  | T7 | 5 | 0.24 | 0.36 | 0.016 | 0 |  |
| **C3** | **3** | **0.23** | **0.19** | **0.015** | **2** |  | C3 | 4 | 0.24 | 0.25 | 0.015 | 0 |  | C3 | 4 | 0.27 | 0.17 | 0.023 | 1 |  |
| Cz | 4 | 0.38 | 0.28 | 0.062**^†^** | 1 |  | Cz | 6 | 0.24 | 0.34 | 0.012 | 0 |  | Cz | 5 | 0.24 | 0.24 | 0.007 | 1 |  |
| C4 | 5 | 0.47 | 0.31 | 0.065**^†^** | 1 |  | C4 | 4 | 0.17 | 0.45 | 0.018 | 1 |  | C4 | 4 | 0.32 | 0.29 | 0.017 | 0 |  |
| T8 | 5 | 0.38 | 0.26 | 0.037 | 0 |  | T8 | 7 | 0.44 | 0.27 | 0.018 | 0 |  | T8 | 4 | 0.24 | 0.25 | 0.019 | 0 |  |
| **CP5** | **5** | **0.25** | **0.18** | **0.045** | **2** |  | CP5 | 5 | 0.36 | 0.20 | 0.029 | 1 |  | CP5 | 5 | 0.22 | 0.20 | 0.021 | 1 |  |
| CP1 | 4 | 0.52 | 0.34 | 0.022 | 0 |  | CP1 | 7 | 0.23 | 0.21 | 0.032 | 1 |  | CP1 | 5 | 0.26 | 0.27 | 0.046**^†^** | 1 |  |
| CP2 | 6 | 0.34 | 0.21 | 0.031 | 1 |  | CP2 | 7 | 0.26 | 0.29 | 0.065^†^ | 1 |  | CP2 | 5 | 0.21 | 0.21 | 0.019 | 1 |  |
| CP6 | 4 | 0.40 | 0.29 | 0.038 | 0 |  | CP6 | 6 | 0.31 | 0.49 | 0.035 | 0 |  | CP6 | 7 | 0.28 | 0.51 | 0.048**^†^** | 1 |  |
| P7 | 5 | 0.39 | 0.28 | 0.065**^†^** | 1 |  | P7 | 6 | 0.27 | 0.25 | 0.034 | 0 |  | P7 | 7 | 0.33 | 0.32 | 0.028 | 0 |  |
| P3 | 7 | 0.38 | 0.41 | 0.046 | 1 |  | **P3** | **8** | **0.29** | **0.26** | **0.051^†^** | **2** |  | P3 | 7 | 0.35 | 0.44 | 0.022 | 0 |  |
| Pz | 6 | 0.49 | 0.17 | 0.049 | 1 |  | Pz | 6 | 0.29 | 0.31 | 0.038 | 0 |  | Pz | 8 | 0.21 | 0.30 | 0.030 | 1 |  |
| P4 | 7 | 0.43 | 0.49 | 0.031**^†^** | 1 |  | P4 | 7 | 0.35 | 0.39 | 0.031 | 0 |  | P4 | 7 | 0.32 | 0.62 | 0.046**^†^** | 1 |  |
| P8 | 6 | 0.38 | 0.25 | 0.059**^†^** | 1 |  | **P8** | **7** | **0.22** | **0.23** | **0.040^†^** | **2** |  | **P8** | **10** | **0.21** | **0.36** | **0.038^†^** | **2** |  |
| POz | 5 | 0.42 | 0.25 | 0.064 | 1 |  | **POz** | **8** | **0.22** | **0.27** | **0.059^†^** | **3** |  | POz | 7 | 0.17 | 0.28 | 0.023 | 1 |  |
| O1 | 8 | 0.40 | 0.31 | 0.038 | 1 |  | O1 | 8 | 0.32 | 0.25 | 0.032 | 1 |  | **O1** | **8** | **0.20** | **0.41** | **0.023** | **2** |  |
| O2 | 8 | 0.48 | 0.50 | 0.057 | 1 |  | **O2** | **9** | **0.31** | **0.35** | **0.061^†^** | **2** |  | O2 | 8 | 0.37 | 0.40 | 0.030 | 1 |  |

Table S2. Alpha band. Hub identification according to the four graph metrics of the degree (D), path length (PL), clustering coefficient (CC) and betweenness centrality (BC);
 for each node and group: GB&W (left), CCI (middle), WCI (right), respectively. — symbol † denotes BC nodes. Bold rows are represented the identified brain hubs with hub scores, HS ≥ 2. In the alpha band, CCI group also showed higher number of hubs compared to WCI and GB&W groups.

| **GB&W (Group 1)** | | | | | |  | **CCI (Group 2)** | | | | | |  | **WCI (Group 3)** | | | | | |
| --- | --- | --- | --- | --- | --- | --- | --- | --- | --- | --- | --- | --- | --- | --- | --- | --- | --- | --- | --- |
| **Nodes** | **D** | **PL** | **CC** | **BC** | **HS** |  | **Nodes** | **D** | **PL** | **CC** | **BC** | **HS** |  | **Nodes** | **D** | **PL** | **CC** | **BC** | **HS** |
| FP1 | 9 | 0.49 | 0.59 | 0.009 | 1 |  | FP1 | 8 | 0.58 | 0.39 | 0.033 | 1 |  | FP1 | 8 | 0.45 | 0.51 | 0.021 | 0 |
| FPz | 9 | 0.47 | 0.41 | 0.015 | 1 |  | FPz | 10 | 0.46 | 0.36 | 0.050 | 1 |  | FPz | 10 | 0.47 | 0.57 | 0.009 | 1 |
| FP2 | 8 | 0.40 | 0.66 | 0.016 | 0 |  | **FP2** | **8** | **0.53** | **0.25** | **0.041** | **2** |  | FP2 | 10 | 0.54 | 0.57 | 0.024 | 1 |
| F7 | 7 | 0.33 | 0.45 | 0.034 | 0 |  | F7 | 8 | 0.49 | 0.34 | 0.026 | 1 |  | F7 | 7 | 0.58 | 0.40 | 0.022 | 0 |
| F3 | 7 | 0.45 | 0.58 | 0.033 | 0 |  | F3 | 6 | 0.40 | 0.34 | 0.021 | 1 |  | F3 | 8 | 0.54 | 0.54 | 0.030 | 0 |
| Fz | 10 | 0.47 | 0.45 | 0.020 | 1 |  | Fz | 7 | 0.50 | 0.31 | 0.025 | 0 |  | Fz | 9 | 0.41 | 0.44 | 0.017 | 0 |
| F4 | 8 | 0.39 | 0.28 | 0.019 | 1 |  | F4 | 6 | 0.45 | 0.34 | 0.017 | 0 |  | F4 | 9 | 0.46 | 0.35 | 0.025 | 1 |
| F8 | 8 | 0.27 | 0.46 | 0.039 | 1 |  | F8 | 8 | 0.43 | 0.36 | 0.022 | 1 |  | **F8** | **7** | **0.34** | **0.28** | **0.023** | **2** |
| FC5 | 6 | 0.24 | 0.33 | 0.007 | 1 |  | FC5 | 6 | 0.34 | 0.30 | 0.034 | 1 |  | FC5 | 5 | 0.56 | 0.38 | 0.016 | 0 |
| **FC1** | **9** | **0.44** | **0.49** | **0.048^†^** | **2** |  | FC1 | 8 | 0.45 | 0.43 | 0.046 | 1 |  | FC1 | 7 | 0.56 | 0.45 | 0.044 | 0 |
| **FC2** | **10** | **0.41** | **0.51** | **0.049^†^** | **2** |  | **FC2** | **10** | **0.54** | **0.35** | **0.067^†^** | **2** |  | FC2 | 9 | 0.44 | 0.56 | 0.030 | 0 |
| FC6 | 6 | 0.31 | 0.41 | 0.018 | 0 |  | FC6 | 4 | 0.46 | 0.43 | 0.041 | 0 |  | FC6 | 7 | 0.40 | 0.45 | 0.030 | 0 |
| T7 | 8 | 0.43 | 0.30 | 0.025 | 1 |  | T7 | 6 | 0.41 | 0.38 | 0.033 | 1 |  | T7 | 7 | 0.56 | 0.43 | 0.025 | 0 |
| C3 | 7 | 0.39 | 0.32 | 0.037 | 1 |  | C3 | 6 | 0.67 | 0.36 | 0.061**^†^** | 1 |  | C3 | 8 | 0.36 | 0.46 | 0.037 | 0 |
| **Cz** | **9** | **0.27** | **0.39** | **0.033** | **2** |  | **Cz** | **8** | **0.59** | **0.38** | **0.057^†^** | **2** |  | **Cz** | **10** | **0.45** | **0.54** | **0.091^†^** | **2** |
| C4 | 7 | 0.35 | 0.46 | 0.026 | 0 |  | **C4** | **6** | **0.35** | **0.29** | **0.044** | **2** |  | C4 | 8 | 0.28 | 0.50 | 0.036 | 1 |
| T8 | 8 | 0.35 | 0.47 | 0.030 | 0 |  | T8 | 7 | 0.62 | 0.29 | 0.025 | 0 |  | **T8** | **8** | **0.46** | **0.35** | **0.072^†^** | **2** |
| CP5 | 7 | 0.58 | 0.32 | 0.040 | 1 |  | **CP5** | **7** | **0.39** | **0.28** | **0.032** | **2** |  | CP5 | 6 | 0.43 | 0.50 | 0.027 | 0 |
| CP1 | 7 | 0.30 | 0.40 | 0.020 | 1 |  | CP1 | 7 | 0.54 | 0.39 | 0.030 | 0 |  | **CP1** | **10** | **0.40** | **0.59** | **0.082^†^** | **2** |
| CP2 | 8 | 0.29 | 0.50 | 0.046 | 1 |  | CP2 | 9 | 0.56 | 0.34 | 0.049 | 1 |  | CP2 | 8 | 0.56 | 0.51 | 0.069**^†^** | 1 |
| CP6 | 7 | 0.48 | 0.55 | 0.027 | 0 |  | CP6 | 7 | 0.49 | 0.24 | 0.039 | 1 |  | CP6 | 8 | 0.45 | 0.36 | 0.048 | 1 |
| P7 | 7 | 0.35 | 0.39 | 0.042 | 0 |  | **P7** | **8** | **0.51** | **0.27** | **0.045** | **2** |  | P7 | 8 | 0.30 | 0.42 | 0.035 | 1 |
| P3 | 7 | 0.56 | 0.39 | 0.047 | 1 |  | **P3** | **8** | **0.48** | **0.32** | **0.054^†^** | **2** |  | P3 | 6 | 0.45 | 0.31 | 0.019 | 1 |
| **Pz** | **9** | **0.27** | **0.48** | **0.060^†^** | **3** |  | Pz | 7 | 0.44 | 0.29 | 0.057**^†^** | 1 |  | **Pz** | **10** | **0.31** | **0.50** | **0.070^†^** | **3** |
| **P4** | **8** | **0.44** | **0.25** | **0.069^†^** | **2** |  | P4 | 6 | 0.46 | 0.31 | 0.022 | 0 |  | **P4** | **10** | **0.42** | **0.42** | **0.053^†^** | **2** |
| **P8** | **6** | **0.37** | **0.27** | **0.048^†^** | **2** |  | **P8** | **9** | **0.35** | **0.35** | **0.011** | **2** |  | P8 | 8 | 0.38 | 0.47 | 0.032 | 0 |
| POz | 8 | 0.34 | 0.62 | 0.055**^†^** | 1 |  | POz | 4 | 0.69 | 0.36 | 0.052**^†^** | 1 |  | POz | 9 | 0.26 | 0.55 | 0.034 | 1 |
| O1 | 4 | 0.40 | 0.42 | 0.044 | 0 |  | O1 | 5 | 0.56 | 0.27 | 0.023 | 1 |  | **O1** | **5** | **0.29** | **0.28** | **0.009** | **2** |
| O2 | 8 | 0.42 | 0.57 | 0.030 | 0 |  | O2 | 7 | 0.44 | 0.37 | 0.011 | 0 |  | O2 | 7 | 0.40 | 0.38 | 0.042 | 0 |

Table S3. Beta band. Hub identification according to the four graph metrics of the degree (D), path length (PL), clustering coefficient (CC) and betweenness centrality (BC);
 for each node and group: GB&W (left), CCI (middle), WCI (right), respectively. — symbol † denotes BC nodes. Bold rows are represented the identified brain hubs with hub scores, HS ≥ 2. In the beta band, WCI group also showed higher number of hubs compared to CCI and GB&W groups.

| **GB&W (Group 1)** | | | | | |  | **CCI (Group 2)** | | | | | |  | **WCI (Group 3)** | | | | | |
| --- | --- | --- | --- | --- | --- | --- | --- | --- | --- | --- | --- | --- | --- | --- | --- | --- | --- | --- | --- |
| **Nodes** | **D** | **PL** | **CC** | **BC** | **HS** |  | **Nodes** | **D** | **PL** | **CC** | **BC** | **HS** |  | **Nodes** | **D** | **PL** | **CC** | **BC** | **HS** |
| FP1 | 11 | 0.34 | 0.31 | 0.027 | 1 |  | **FP1** | **9** | **0.52** | **0.28** | **0.028** | **2** |  | FP1 | **12** | 0.41 | 0.25 | 0.024 | 1 |
| **FPz** | **11** | **0.41** | **0.42** | **0.027** | **2** |  | **FPz** | **10** | **0.53** | **0.40** | **0.038^†^** | **4** |  | FPz | **12** | 0.38 | 0.25 | 0.022 | 1 |
| FP2 | 8 | 0.36 | 0.32 | 0.028 | 0 |  | FP2 | 8 | 0.38 | 0.34 | 0.028 | 0 |  | **FP2** | **9** | **0.58** | **0.36** | **0.012** | **2** |
| **F7** | **11** | **0.32** | **0.41** | **0.060^†^** | **2** |  | **F7** | **8** | **0.55** | **0.40** | **0.023** | **2** |  | F7 | **9** | 0.32 | 0.32 | 0.015 | 1 |
| F3 | 7 | 0.33 | 0.36 | 0.023 | 0 |  | F3 | 8 | 0.44 | 0.37 | 0.025 | 0 |  | F3 | 6 | 0.18 | 0.34 | 0.013 | 0 |
| **Fz** | **13** | **0.26** | **0.42** | **0.029^†^** | **2** |  | **Fz** | **9** | **0.44** | **0.41** | **0.027** | **2** |  | **Fz** | **9** | **0.53** | **0.27** | **0.017** | **2** |
| F4 | 8 | 0.27 | 0.35 | 0.011 | 0 |  | F4 | 6 | 0.39 | 0.22 | 0.016 | 0 |  | **F4** | **7** | **0.30** | **0.41** | **0.048^†^** | **2** |
| F8 | 8 | 0.27 | 0.42 | 0.022 | 0 |  | F8 | **10** | 0.47 | 0.29 | 0.030 | 1 |  | F8 | 8 | 0.43 | 0.27 | 0.010 | 0 |
| FC5 | 6 | 0.33 | 0.40 | 0.011 | 0 |  | FC5 | 6 | 0.34 | 0.36 | 0.021 | 0 |  | FC5 | 4 | 0.31 | 0.35 | 0.003 | 0 |
| FC1 | 10 | 0.33 | 0.50 | 0.020 | 1 |  | FC1 | 7 | 0.28 | 0.36 | **0.045^†^** | 1 |  | **FC1** | **9** | **0.46** | **0.27** | **0.038^†^** | **3** |
| FC2 | 9 | 0.30 | 0.36 | 0.019 | 0 |  | FC2 | **9** | 0.43 | 0.30 | 0.037 | 1 |  | FC2 | 7 | 0.34 | 0.34 | 0.030 | 0 |
| FC6 | 5 | 0.29 | 0.38 | 0.013 | 0 |  | FC6 | 6 | **0.57** | 0.27 | 0.022 | 1 |  | FC6 | 6 | 0.28 | 0.33 | 0.005 | 0 |
| T7 | 10 | 0.30 | 0.34 | 0.027 | 0 |  | T7 | 6 | 0.42 | 0.26 | 0.019 | 0 |  | T7 | 6 | 0.40 | 0.30 | 0.022 | 0 |
| C3 | 8 | 0.24 | 0.39 | 0.021 | 0 |  | C3 | 6 | 0.31 | 0.28 | 0.026 | 0 |  | C3 | 8 | 0.21 | 0.29 | 0.020 | 0 |
| Cz | 8 | 0.36 | 0.39 | 0.014 | 0 |  | Cz | 6 | 0.41 | 0.28 | 0.028 | 0 |  | **Cz** | **11** | **0.36** | **0.32** | **0.036^†^** | **2** |
| C4 | 7 | 0.35 | 0.41 | 0.023 | 0 |  | C4 | 7 | 0.29 | 0.30 | 0.015 | 0 |  | C4 | **9** | 0.35 | 0.36 | 0.029 | 1 |
| T8 | 5 | 0.40 | 0.32 | 0.015 | 1 |  | T8 | 6 | 0.44 | 0.30 | 0.015 | 0 |  | T8 | 7 | 0.38 | 0.31 | 0.022 | 0 |
| CP5 | 8 | 0.37 | 0.35 | 0.020 | 0 |  | CP5 | 7 | **0.47** | 0.27 | 0.037 | 1 |  | CP5 | 6 | 0.37 | **0.39** | 0.014 | 1 |
| CP1 | 7 | 0.23 | 0.38 | 0.018 | 0 |  | **CP1** | **9** | **0.40** | **0.33** | **0.047^†^** | **2** |  | **CP1** | **11** | **0.48** | **0.40** | **0.045^†^** | **4** |
| **CP2** | **9** | **0.41** | **0.51** | **0.044^†^** | **3** |  | **CP2** | **9** | **0.39** | **0.38** | **0.037^†^** | **3** |  | **CP2** | **9** | **0.36** | **0.40** | **0.058^†^** | **3** |
| CP6 | 6 | 0.28 | 0.36 | 0.020 | 0 |  | CP6 | 8 | 0.38 | 0.30 | 0.014 | 0 |  | CP6 | 8 | 0.31 | 0.30 | 0.023 | 0 |
| **P7** | **8** | **0.39** | **0.42** | **0.038^†^** | **2** |  | P7 | 8 | 0.44 | 0.36 | **0.055^†^** | 1 |  | **P7** | **9** | **0.47** | **0.32** | **0.015** | **2** |
| P3 | 10 | 0.34 | 0.37 | 0.029 | 0 |  | P3 | 7 | 0.42 | 0.32 | 0.023 | 0 |  | P3 | **12** | 0.41 | 0.34 | 0.033 | 1 |
| **Pz** | **11** | **0.47** | **0.42** | **0.035^†^** | **4** |  | **Pz** | **8** | **0.45** | **0.38** | **0.040^†^** | **2** |  | Pz | 8 | 0.16 | 0.37 | 0.022 | 0 |
| P4 | 9 | 0.39 | 0.39 | 0.027 | 0 |  | P4 | 7 | 0.33 | 0.30 | 0.019 | 0 |  | P4 | 9 | 0.33 | **0.42** | 0.023 | 1 |
| P8 | 7 | 0.45 | 0.28 | 0.024 | 1 |  | P8 | **9** | 0.42 | 0.35 | 0.030 | 1 |  | P8 | 8 | **0.48** | 0.35 | 0.023 | 1 |
| **POz** | **11** | **0.49** | **0.41** | **0.035^†^** | **3** |  | POz | **9** | 0.39 | 0.35 | 0.036 | 1 |  | **POz** | **9** | **0.36** | **0.25** | **0.047^†^** | **2** |
| O1 | 7 | 0.37 | 0.42 | 0.017 | 1 |  | O1 | 7 | **0.49** | 0.31 | 0.035 | 1 |  | **O1** | **9** | **0.43** | **0.42** | **0.023** | **2** |
| O2 | 7 | 0.32 | 0.42 | 0.020 | 1 |  | O2 | 8 | 0.40 | **0.37** | 0.024 | 1 |  | O2 | 7 | 0.38 | 0.31 | 0.022 | 0 |

**References**

Bunzeck, N., Guitart-Masip, M., Dolan, R. J., & Düzel, E. (2011). Contextual novelty modulates the neural dynamics of reward anticipation. *Journal of Neuroscience, 31*(36), 12816-12822.

Bush, G., Luu, P., & Posner, M. I. (2000). Cognitive and emotional influences in anterior cingulate cortex. *Trends in Cognitive Sciences, 4*(6), 215-222.

Harmony, T. (2013). The functional significance of delta oscillations in cognitive processing. *Frontiers in integrative neuroscience, 7*, 83.

Howard, M. W., Rizzuto, D. S., Caplan, J. B., Madsen, J. R., Lisman, J., Aschenbrenner-Scheibe, R., . . . Kahana, M. J. (2003). Gamma oscillations correlate with working memory load in humans. *Cerebral Cortex, 13*(12), 1369-1374.

Jensen, O., & Tesche, C. D. (2002). Frontal theta activity in humans increases with memory load in a working memory task. *European Journal of Neuroscience, 15*(8), 1395-1399.

Klimesch, W. (1996). Memory process, brain oscillations and EEG synchronization. *International Journal of Psychophysiology, 24*(1-2), 61-100.

Klimesch, W. (1999). EEG alpha and theta oscillations reflect cognitive and memory performance_A review and analysis. *Brain Research Reviews, 29*, 169–195.

Kortelainen, J., Väyrynen, E., & Seppänen, T. (2015). High-frequency electroencephalographic activity in left temporal area is associated with pleasant emotion induced by video clips. *Computational intelligence and neuroscience, 2015*, 31.

Ray, W. J., & Cole, H. W. (1985). EEG alpha activity reflects attentional demands, and beta activity reflects emotional and cognitive processes. *Science, 228*(4700), 750-752.

Rowland, N., Meile, M., & Nicolaidis, S. (1985). EEG alpha activity reflects attentional demands, and beta activity reflects emotional and cognitive processes. *Science, 228*(4700), 750-752.

Sammler, D., Grigutsch, M., Fritz, T., & Koelsch, S. (2007). Music and emotion: electrophysiological correlates of the processing of pleasant and unpleasant music. *Psychophysiology, 44*(2), 293-304.

Scholz, S., Schneider, S. L., & Rose, M. (2017). Differential effects of ongoing EEG beta and theta power on memory formation. *PloS one, 12*(2), e0171913.

Von Stein, A., Chiang, C., & König, P. (2000). Top-down processing mediated by interareal synchronization. *Proceedings of the National Academy of Sciences, 97*(26), 14748-14753.

Welch, P. D. (1967). The use of fast Fourier transform for the estimation of power spectra: A method based on time averaging over short, modiﬁed periodograms. *IEEE Transactions on audio and electroacoustics, 15*(2), 70-73.
