# Supplementary figures and images for: Exploring EEG Effective Connectivity Network in Estimating Influence of Color on Emotion and Memory
Source: Front Neuroinform. 2019 Oct 9;13:66. doi: 10.3389/fninf.2019.00066 (PMC6794354; doi:10.3389/fninf.2019.00066)

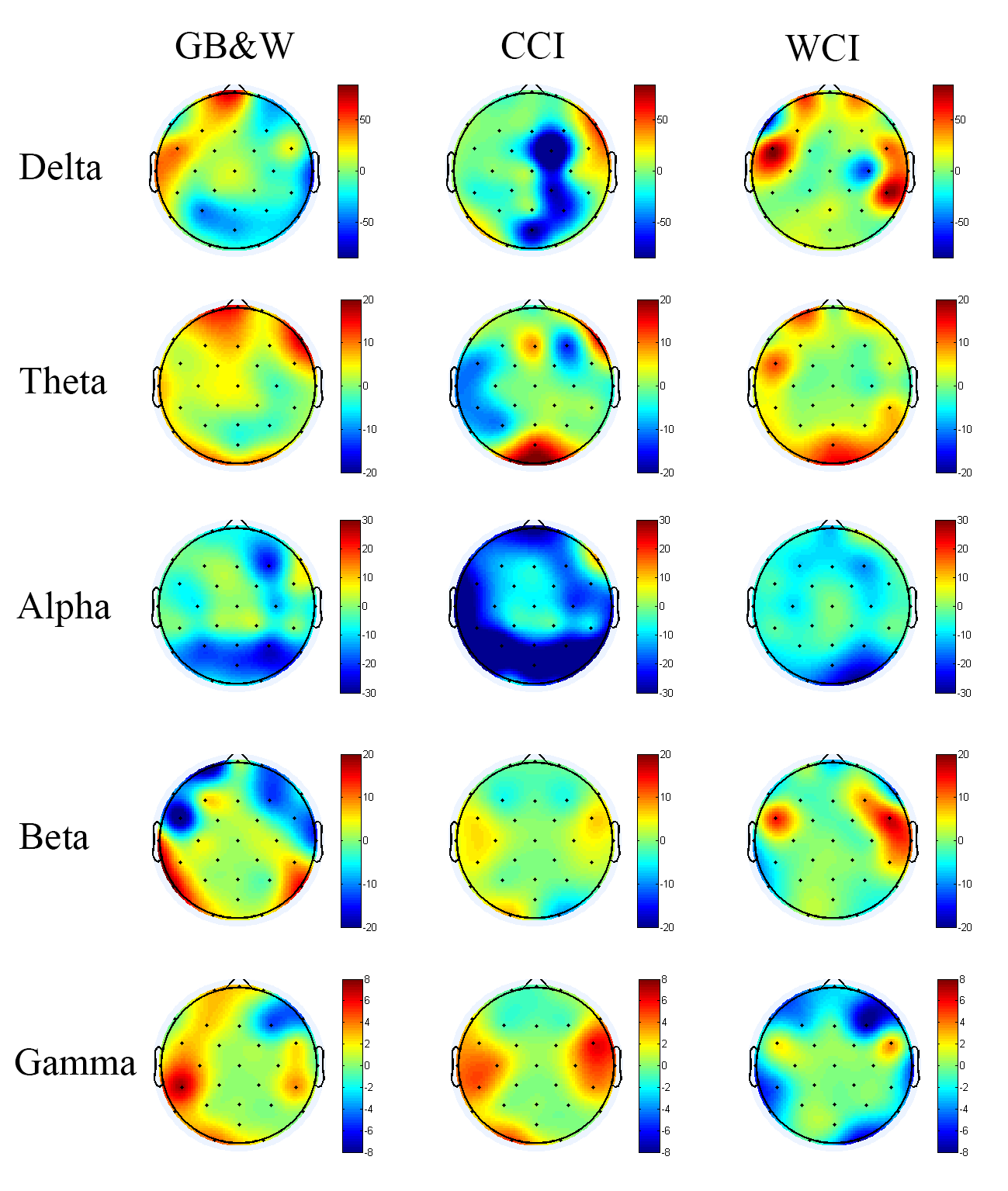

Supplement: Supplementary file 2 [file Image_1.TIF]
